# Gastric squamous-columnar junction contains a large pool of cancer-prone immature osteopontin responsive Lgr5^−^CD44^+^ cells

**DOI:** 10.1038/s41467-019-13847-2

**Published:** 2020-01-03

**Authors:** Dah-Jiun Fu, Lianghai Wang, Fouad K. Chouairi, Ian M. Rose, Danysh A. Abetov, Andrew D. Miller, Robert J. Yamulla, John C. Schimenti, Andrea Flesken-Nikitin, Alexander Yu. Nikitin

**Affiliations:** 1 Department of Biomedical Sciences and Cornell Stem Cell Program, Cornell University, Ithaca, NY 14853 USA; 2 Department of Pathology and Key Laboratories for Xinjiang Endemic and Ethnic Diseases, Shihezi University School of Medicine, Shihezi, Xinjiang China

**Keywords:** Gastric cancer, Adult stem cells

## Abstract

Areas of a junction between two types of epithelia are known to be cancer-prone in many organ systems. However, mechanisms for preferential malignant transformation at the junction areas remain insufficiently elucidated. Here we report that inactivation of tumor suppressor genes *Trp53* and *Rb1* in the gastric squamous-columnar junction (SCJ) epithelium results in preferential formation of metastatic poorly differentiated neoplasms, which are similar to human gastroesophageal carcinoma. Unlike transformation-resistant antral cells, SCJ cells contain a highly proliferative pool of immature Lgr5^−^CD44^+^ cells, which are prone to transformation in organoid assays, comprise early dysplastic lesions, and constitute up to 30% of all neoplastic cells. CD44 ligand osteopontin (OPN) is preferentially expressed in and promotes organoid formation ability and transformation of the SCJ glandular epithelium. OPN and CD44 overexpression correlate with the worst prognosis of human gastroesophageal carcinoma. Thus, detection and selective targeting of the active OPN-CD44 pathway may have direct clinical relevance.

## Abstract (web summary)

Cancers arising from the gastric squamous-columnar junction have high incidence and are characterized by a poor prognosis. Here, the authors use genetic mouse models to show that loss of p53 and Rb1 expression results in preferential tumour development at the gastric squamous-columnar junction that contains a large pool of osteopontin responsive Lgr5-CD44+ cells.

## Introduction

Gastric cancer is the fifth most common malignancy and the third leading cause of cancer mortality with an estimated 723,000 deaths (8.8% of total) worldwide^1,2^. Due to improvements of dietary hygiene and decline of *Helicobactor pylori* infection, the incidence rate of gastric cancer has decreased by more than 80% since 1950s^3,4^. Unfortunately, the incidence of cancers arising from the gastric squamous-columnar junction (SCJ, aka gastroesophageal junction), the area of a direct transition from the esophageal stratified squamous epithelium to the gastric glandular epithelium, has steadily increased^5,6^. The incidence of gastric SCJ cancer has risen nearly 2.5-fold in the United States from 1970s to 2000s, being responsible for approximately half of all gastric cancer cases in 2008^6^. Notably, the prognosis of the gastric SCJ cancer is generally worse than cancers located in other regions of the stomach. The 5-year survival rate of the patients with gastric SCJ cancer is ~2–12%, compared with 20–25% for all gastric cancers^6,7^. The underlying reasons for the increase in SCJ cancer frequency and poorer prognosis remain unknown.

Since SCJ carcinomas frequently span the SCJ^6^, the accurate demarcation of their origin remains challenging. Recent comprehensive genomic studies suggest that esophageal adenocarcinomas and gastric adenocarcinomas of the chromosomally unstable subtype, which are predominantly located in SCJ/cardia, may represent closely related but not identical disease entities^8^.

Numerous studies have suggested that epithelial transitional zones (TZs, aka, epithelial junctions) are more predisposed to cancer than other regions in the same organ^9–13^. During recent years, it has been recognized that many TZs contain stem cell niches responsible for the tissue regeneration and repair upon injury. Previous studies have shown that such niches may be particularly prone to the malignant transformation. Such examples include TZ in the mouse ovarian hilum region^9,14^ and human tubal-peritoneal junction^15^. However, the applicability of these observations to TZs in other organs remains uncertain. Furthermore, the mechanisms responsible for preferential susceptibility to cancer by TZ stem cells, as opposed to those in other regions of the same organ, remain insufficiently understood.

In mice, SCJ divides squamous and glandular regions of the stomach. It is commonly accepted that mouse SCJ represents an appropriate equivalent for studies of human SCJ which is TZ between the esophagus and stomach^16–18^. Several genetically modified mouse models have been developed to study Barrett’s esophagus, which is defined by the replacement of esophageal stratified squamous epithelium with intestinal-like columnar epithelium at the distal end of the esophagus. Barrett’s esophagus is considered to be a precursor lesion associated with the initiation of low-grade dysplasia, high-grade dysplasia, and adenocarcinoma in the SCJ^11^. A number of alternative putative cells of origin of Barrett’s esophagus has been proposed, such as embryonic residual cells in the SCJ^19^, the transdifferentiated squamous epithelial cells of the esophagus^20,21^, the subpopulation of esophageal basal stem cells^22^, the submucosal gland of esophagus^23^, the circulating bone marrow progenitor cells^24^, the cardia glandular epithelial cells^11^, and the transitional basal cells at the SCJ^25^. Unfortunately, none of the above experimental models provide direct evidence that Barrett’s esophagus-like lesions derived from these cellular candidates can progress to advanced metastatic malignancy. Furthermore, the cell of origin of SCJ gastric cancers, which do not progress through Barrett’s esophagus-like lesions, remains uncertain.

A broad spectrum of mutations has been reported to be involved in the carcinogenesis of human gastric SCJ^26,27^. According to genome-wide studies, mutations of *TP53* gene are observed in 70–83% of gastroesophageal cancers^8,26,28,29^. At the same time, over 72% of these cancers contain aberrations in components of RB1 pathway, such as *CDKN2A* (32–81%), *CDK4* (3%), *CCND1* (10–15%), *CCNE1* (12–14%), *CDK6* 14–17%), and *RB1* (1–5%)^8,26,27,29–31^

In this study, we establish a mouse model for metastatic gastric SCJ carcinoma that is based on conditional inactivation of *Trp53* and *Rb1* in Lgr5^+^ cells. We identify a distinct cancer-prone immature Lgr5^−^CD44^+^ population in the first gland of gastric SCJ area. We also provide several lines of evidence supporting the critical role of osteopontin (OPN)-CD44 signaling in the initiation and progression of gastric SCJ cancers.

## Results

### Preferential cancer susceptibility of the gastric SCJ

Lgr5^+^ cells are present in multiple tissues, including TZs, such as gastric SCJ and ovarian hilum^14,32,33^ (Supplementary Fig. 1). To test if Lgr5^+^ stem cells located in TZs are more susceptible to the malignant transformation as compared with their counterparts in other areas of the same tissue we have crossed *Lgr5*^*tm1(cre/ERT2)Cle*^*/J* (*Lgr5*^*eGFP*−*Ires*−*CreERT*^) mice, which harbor an Lgr5-eGFP-IRES-CreERT2 knock in allele, with mice carrying conditional alleles of *Trp53* and *Rb1* (aka *p53/TP53* and *Rb/RB1*), respectively. To trace cells exposed to tamoxifen-activated Cre-ERT2 fusion protein mice were also crossed to *Rosa-loxP-stop-loxP-tdTomato* (Ai9) reporter mice (Supplementary Fig. 2a).

**Fig. 1:**
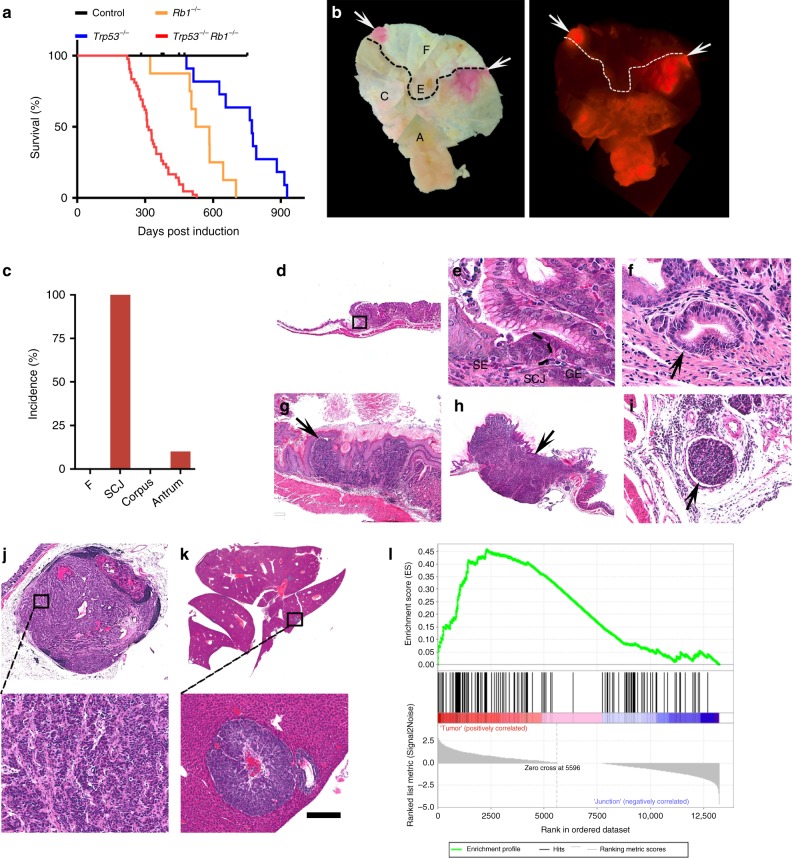
Inactivation of *Trp53* and *Rb1* in Lgr5^+^ cells leads to preferential neoplasia at the gastric SCJ. **a** Kaplan–Meier survival curves of *Lgr5*^*eGFP*−*Ires*−*CreERT2*^*Trp53*^*loxP/loxP*^*Rb1*^*loxP/loxP*^Ai9 mice with (*Trp53*^−/−^*Rb1*^−/−^, *n* = 42) and without (Control, *n* = 9) tamoxifen treatment (log-rank test *P* < 0.0001), and *Lgr5*^*eGFP*−*Ires*−*CreERT2*^*Trp53*^*loxP/loxP*^Ai9 (*Trp53*^−/−^, *n* = 11) mice and *Lgr5*^*eGFP*−*Ires*−*CreERT2*^*Rb1*^*loxP/loxP*^Ai9 mice (*Rb1*^−/−^, *n* = 8) treated with tamoxifen (*P* < 0.001 vs *Trp53*^−/−^*Rb1*^−/−^ mice for both *Trp53*^−/−^ and *Rb1*^−/−^). **b** tdTomato-expressing (tdTomato^+^) neoplastic masses (red, arrows) in the stomach of *Lgr5*^*eGFP*−*Ires*−*CreERT2*^*Trp53*^*loxP/loxP*^*Rb1*^*loxP/loxP*^ Ai9 mouse 291 days post induction (p.i.) with tamoxifen. Bright field (left) and fluorescence (right). The punctate lines indicate SCJ line. F forestomach, E esophagus, C corpus, A antrum. **c** Distribution of neoplasms in stomach regions (*n* = 20). **d**, **e** SCJ between the squamous (SE) and glandular (GE) epithelium in adult *Lgr5*^*eGFP*−*Ires*−*CreERT2*^*Trp53*^*loxP/loxP*^*Rb1*^*loxP/loxP*^Ai9 mouse without tamoxifen induction. Rectangle in **d** indicates area shown in **e**. SCJ carcinogen**e**sis in *Lgr5*^*eGFP*−*Ires*−*CreERT2*^*Trp53*^*loxP/loxP*^*Rb1*^*loxP/loxP*^Ai9 mice includes dysplastic glandular lesions (**f**, arrow, 120 days p.i.), early carcinoma (**g**, arrow, 177 days p.i.), and advanced carcinoma (**h**, arrow, 239 days p.i.) with vascular invasion (**i**). Preferent**i**al location of SCJ carcinoma metastases in the lymph node (**j**, 20% of cases) and the liver (**k** 35% of cases). Rectangle in top images indicates areas shown in bottom images. **l** Gene Set Enrichment Analysis of RNA-seq data of mouse gastric SCJ cancers (*n* = 5) using human gastroesophageal cancer signature genes^70^. Enrichment score (ES) = 0.46, normalized enrichment score (NES) = 1.38, false discovery rate (FDR) *q* value = 0.08, *P* < 0.05. **d–k** Hematoxylin and eosin staining. Scale bar in **k** represents 3 mm (**b**), 1 mm (**d** and **h**), 60 μm (**e**), 40 μm (**f**), 300 μm (**g**), 100 μm (**i**), 600 μm (**j** top panel), 100 μm (**j** bottom panel), 4.5 mm (**k** top panel), and 175 μm (**k** bottom panel). Source data are provided as a Source Data file.

All mice succumbed to poorly differentiated, highly invasive, and metastatic gastric carcinomas with median survival of 312 days after first injection of tamoxifen to 60 days old mice (Fig. 1a, b). Male mice had shorter survival time (median = 239 days) than females (median = 329 days) (Supplementary Fig. 2b). All gastric malignant neoplasms were located within the area of SCJ (Fig. 1c). In addition, 10% (2/20) of mice displayed benign tumors, adenomas, in the antral region (Supplementary Fig. 2c). All females also developed dysplastic lesions in the ovarian hilum, and some mice developed squamous cell carcinomas and adrenal pheochromocytomas (Supplementary Fig. 2c, d). No neoplastic lesions were observed in the intestine or any other evaluated organs. This suggests that either different genetic alterations or longer time for carcinogenesis are required for some tissues. According to previous results^33,34^ and our cell lineage tracing studies (Supplementary Fig. 3), Lgr5^+^ stem cells are responsible for the routine epithelial renewal in the gastric SCJ and antral regions. The antrum contains the majority of Lgr5^+^ stem cells targeted for our mutations (Supplementary Fig. 3), yet cancer incidence was strikingly higher in the SCJ (Fig. 1c).

According to sequential analysis of carcinogenesis in the proximal stomach, early dysplastic epithelial lesions were observed at the bottom to middle part of the first gastric pit (aka first gland) at the SCJ (Fig. 1d–f) by 60 to 120 days post induction (p.i.). Such lesions were characterized by cells with enlarged pleomorphic and hyperchromatic nuclei and irregular shapes. These lesions progressed to poorly differentiated carcinomas (Fig. 1g, h) invading submucosal and muscular layers and vessels (Fig. 1i), and metastasizing to regional lymph nodes (4/20; 20%; Fig. 1j) and liver (7/20; 35%; Fig. 1k, and Supplementary Fig. 2). This metastatic pattern of SCJ cancers, as well as their faster development in male mice, well correlated with similar features in human gastroesophageal carcinoma^35^. Supporting the clinical relevance of our mouse model, the gene set enrichment analyses (GSEA) of the RNA-seq transcriptional profile of the RNA samples isolated from mouse SCJ carcinomas was indicative of concordance with the expression of signature gene set of human gastroesophageal carcinomas (normalized enrichment score = 1.38, *P* < 0.05; Fig. 1l). A number of pathways upregulated in human gastroesophageal cancer were also upregulated in mouse SCJ cancer model (Supplementary Figs. 2e and 4).

### Large fraction of proliferating immature cells at SCJ

**Fig. 2:**
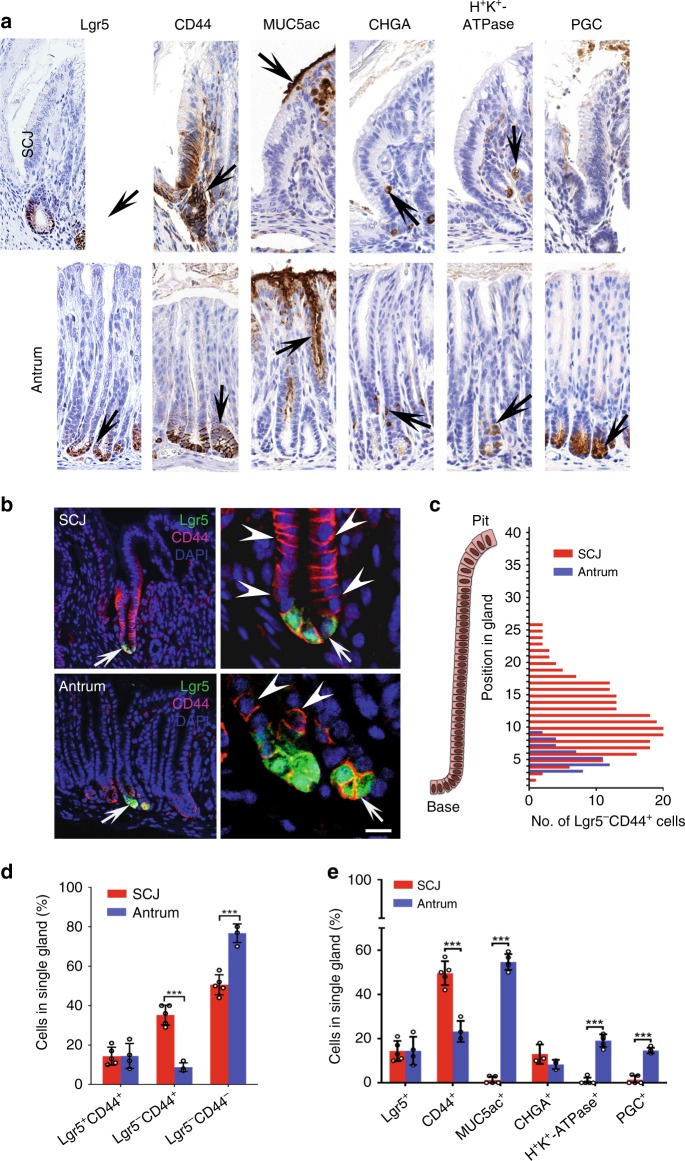
Comparison of cellular components between gastric SCJ and antrum. **a** Detection (brown color, arrows) of cells expressing Lgr5 (Lgr5-eGFP), CD44, and differentiation markers of pit cells (Mucin5AC), neuroendocrine cells (chromogranin A, CHGA), parietal cells (H^+^K^+^-ATPase), and chief cells (pepsinogen C, PGC). **b** Detection of Lgr5-eGFP (green) and CD44 (magenta) in the SCJ and antral epithelium. Arrows in left column images indicate areas shown in right column. Arrows and arrowheads in right column images indicate expression of Lgr5-eGFP and CD44, respectively. **c** Number of Lgr5^−^CD44^+^ cells located at specific positions in glands of SCJ and antrum (*n* = 20 in each group). **d** Quantitative analysis of Lgr5^+^CD44^+^, Lgr5^−^CD44^+^, Lgr5^−^CD44^−^, and OPN^+^ cells at SCJ and antrum (*n* = 20 in each group). **e** Quantification of cells expressing Lgr5 (Lgr5-eGFP), CD44, and differentiation markers (*n* = 3 or more in each group). Elite ABC method (**a**) and immunofluorescence (**b**). Counterstaining with hematoxylin (**a**) and DAPI (**b**). Scale bar in **b** represents 40 μm (**a**), 50 μm (left panels of **b**), 15 μm (upper right panel of **b**), and 10 μm (lower right panel of **b**). **P < 0.01, ***P < 0.001, two-tailed unpaired *t*-tests. All error bars denote s.d. Source data are provided as a Source Data file.

To investigate if the difference in cancer susceptibility can be explained by variations in cellular compositions of the first pit of SCJ and antral glands, we performed serial immunostaining for the following markers: Lgr5-eGFP for stem cells, CD44 for stem/progenitor cells, Mucin5AC for pit cells, H^+^K^+^-ATPase for parietal cells, chromogranin A for neuroendocrine cells, and pepsinogen C for chief cells (Fig. 2a). Notably, a large fraction of first pit CD44^+^ cells (35.2 ± 5.1%) did not express Lgr5, with Lgr5^−^CD44^+^ cells being distributed from the bottom to middle length of the first pit (Fig. 2b–d). Lgr5^−^CD44^+^ SCJ cells were actively proliferating (Supplementary Fig. 5a–c) and remained immature according to the lack of differentiation markers typical for pit, parietal cells, and chief cells (Fig. 2e). On the contrary, all antral CD44^+^ cells were located at the base of the pit, contained smaller fraction of Lgr5^−^CD44^+^ population of cells per single gland (8.75 ± 2.2%; Fig. 2b–d), and showed limited proliferative activity (Supplementary Fig. 5a-c). According to lineage tracing experiments, all Lgr5^−^CD44^+^ cells derived from Lgr5^+^ cells (Supplementary Fig. 5d). Taken together, we identified a uniquely large fraction of immature Lgr5^−^CD44^+^ population in the first pit of gastric SCJ.

### Lgr5^−^CD44^+^ immature cells are prone to transformation

**Fig. 3:**
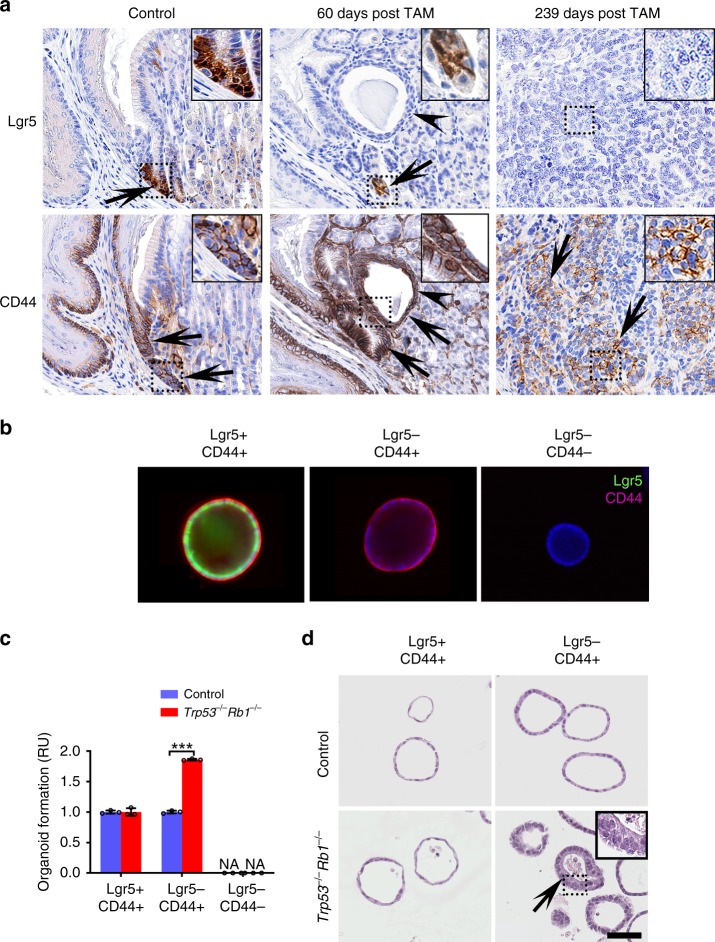
Lgr5^−^CD44^+^ cells of SCJ show preferential malignant transformation. **a** Detection of Lgr5-eGFP (arrows in top panels, and CD44 (arrows in bottom panels) in the gastric SCJ before (Control) and after (60 days post TAM and 239 days post TAM) tamoxifen (TAM) administration to *Lgr5*^*eGFP*−*Ires*−*CreERT2*^*Trp53*^*loxP/loxP*^*Rb1*^*loxP/loxP*^Ai9 mice. Early stage dysplastic lesions (arrowheads, TAM + 60 days) and advanced carcinoma (TAM + 239 days) are shown at the SCJ region. Punctate rectangles indicate areas shown in insets. **b** Gastric SCJ organoids prepared from *Lgr5*^*DTR*−*eGFP*^ (*Lgr5*-DTR) mice and cultured under conditions promoting presence of Lgr5^+^CD44^+^ cells (CHIR99021 and valproic acid), Lgr5^−^CD44^+^ cells (diphtheria toxin), or mature Lgr5^−^CD44^−^ cells (Wnt3a-, FGF-, noggin-, and gastrin- media with diphtheria toxin). Representative immunofluorescence of Lgr5-eGFP (turquoise) and CD44 (magenta) of SCJ organoids. **c** Frequency of gastric SCJ organoids with preferential presence of Lgr5^+^CD44^+^, Lgr5^−^CD44^+^, and Lgr5^−^Cd44^−^ cells before (Control) and after *Trp53* and *Rb1* inactivation (*n* = 3 in each group). NA not available. Normalized to control groups. **d** Morphology of gastric SCJ organoids formed before (Control) and after CRISPR-mediated *Trp53* and *Rb1* inactivation. Arrows indicate dysplastic changes in transformed organoids 11 days (passage 2) after inactivation of *Trp53* and *Rb1*. Rectangle indicates area shown in inset. Elite ABC method, hematoxylin counterstaining (**a**), Immunofluorescence, DAPI counterstaining (**b**), and hematoxylin and eosin (H&E) staining (**d**). Scale bar in **d** represents 60 μm (**a**), 25 μm (**a**, insets), 85 μm (**b**), 55 μm (**d**), and 22 μm (**d**, inset). *P < 0.05, ***P < 0.001, two-tailed unpaired *t*-tests. All error bars denote s.d. Source data are provided as a Source Data file.

To identify the most likely cellular origin of gastric SCJ cancer, we have evaluated expression of Lgr5 and CD44 during carcinogenesis in our *Lgr5*^*eGFP*−*Ires*−*CreERT2*^*Trp53*^*loxP/loxP*^*Rb1*^*loxP/loxP*^Ai9 mouse model. The early lesions were observed at the gastric SCJ region 60 days after exposure to tamoxifen (Fig. 3a). The affected cells showed dysplastic characteristics, such as enlarged pleomorphic and hyperchromatic nuclei with irregular shapes (Fig. 3a). The Lgr5^+^ stem cells were found at the base of morphologically normal first pit of SCJ but no Lgr5 expression was observed in dysplastic lesions (Fig. 3a). At the same time, the majority of dysplastic cells showed prominent CD44 expression. Consistently, CD44 but not Lgr5 expression was observed in the advanced SCJ carcinomas.

To compare the transformation potential among various cell populations in gastric epithelium, we isolated the gastric epithelium cells from the SCJ of *Lgr5*^*DTR*−*eGFP*^ (*Lgr5-DTR*) mice, and cultured them under conditions that promote preferential organoid composition as Lgr5^+^ cells (addition of GSK3β inhibitor CHIR99021, and histone deacetylase inhibitor valproic acid, VPA), Lgr5^−^CD44^+^ cells (ablation of Lgr5^−^ cells by diphtheria toxin treatment), or Lgr5^−^CD44^−^ cells (Wnt3a-, FGF-, noggin-, and gastrin- media accompanied with diphtheria toxin treatment)^36^ (Fig. 3b, and Supplementary Fig. 6a–c). Following clustered regularly interspaced palindromic repeats (CRISPR)/Cas9-mediated *Trp53* and *Rb1* inactivation, the most pronounced changes were observed in Lgr5^−^CD44^+^ organoids. These changes included increase in organoid formation ability, size, and proliferation, and marked alterations in their morphology from a single layer of cuboidal cells to multilayered dysplastic cell structures (Fig. 3c, d and Supplementary Fig. 6c–e). The transformed Lgr5^−^CD44^+^ organoids contained a high fraction of proliferating cells (Supplementary Fig. 7a, b). Treatment of organoids with CHIR99021 and VPA increased expression of *Notch1* and its downstream targets *Hes1*, *Hey1*, and *Hey2* (Supplementary Fig. 7c). Thus, it is unlikely that the lack of dysplastic morphology in Lgr5+/CD44+ organoids can be explained by CHIR99021-mediated downregulation of NOTCH signaling. No organoids, including those with multilayered dysplastic features, stained for KRT5, a marker of the squamous epithelium, thereby confirming their origin from the glandular part of SCJ (Supplementary Fig. 7d, e).

Wild-type control and the *Trp53*^*−/−*^*Rb1*^*−/−*^ gastric organoids were transplanted into the flanks of NSG mice. The *Trp53*^*−/−*^*Rb1*^*−/−*^ organoids gave rise to invasive, poorly differentiated carcinomas starting from 223 days post grafting (Supplementary Fig. 8). These carcinomas expressed tdTomato, confirming their allograft origin, and characterized by predominant CD44 expression and absence of Lgr5 expression (Supplementary Fig. 8). Taken together, these findings indicate that either SCJ carcinoma is formed by Lgr5^−^CD44^+^ cells or Lgr5^+^ stem cells are rapidly outcompeted by Lgr5^−^CD44^+^neoplastic cells at the time of initiation.

### CD44 plays critical role in SCJ epithelium transformation

**Fig. 4:**
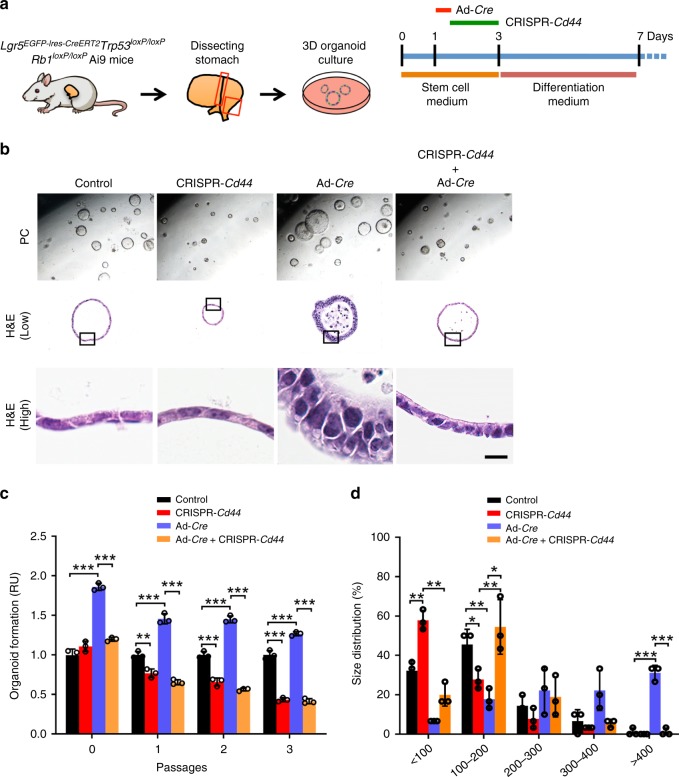
The role of CD44 in the growth and transformation of gastric organoids. **a** Experimental design. The rectangles indicate regions used for organoid preparation. **b** Organoids formed by gastric epithelial cells derived from *Lgr5*^*eGFP*−*Ires*−*CreERT2*^*Trp53*^*loxP/loxP*^*Rb1*^*loxP/loxP*^Ai9 mice before (control) and after deletion of CD44 (CRISPR-*Cd44*), *Trp53*, and *Rb1* (Ad-*Cre*) or all three genes (CRISPR-*Cd44* + Ad-*Cre*). Rectangle in middle row images indicates area shown in bottom row images. The relative numbers (**c**) and sizes (**d**) of gastric organoids before and after inactivation of *Trp53* and *Rb1* (Ad-*Cre*) and/or CD44 (CRISPR-*Cd44*). The organoid numbers were normalized to control organoids at the same passage. The scale bar represents 500 μm (**b**, top panels), 70 μm (**b**, middle panels), and 9 μm (**b**, bottom panels). *P < 0.05, **P < 0.01, ***P < 0.001, two-tailed unpaired *t*-tests. All error bars denote s.d. Source data are provided as a Source Data file.

**Fig. 5:**
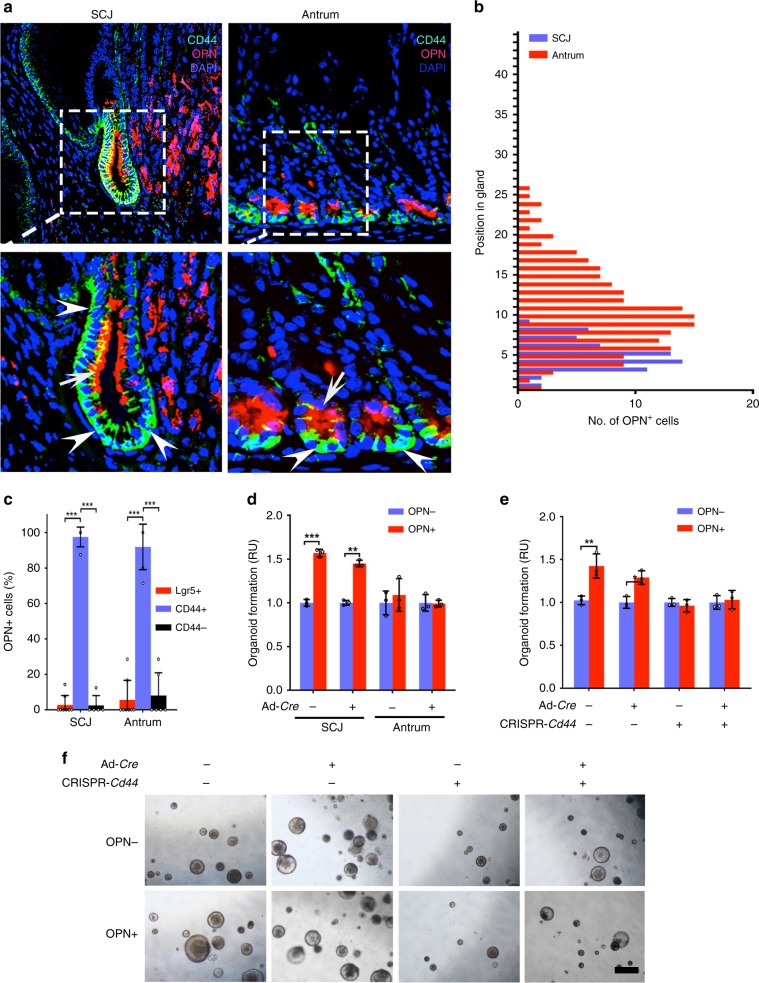
Distribution of the OPN^+^ cells in the gastric SCJ and antrum. **a** Detection of OPN (red, arrows) and CD44 (turquoise, arrowheads) in the SCJ and antrum of wild-type mice. Punctate rectangles in top row images indicate areas shown in the bottom row images. **b** Number of OPN^+^ cells located at specific positions in glands SCJ and antrum (*n* = 20, each). **c** Fractions of the OPN^+^ cells that express Lgr5 or CD44 within the regions as shown (*n* = 05 or more). **d** The relative numbers of gastric organoids before (OPN−) and after (OPN+) exogenous OPN treatment. The organoid numbers were normalized to nontreated control in each group. **e** The relative numbers of SCJ gastric organoids before (OPN−) and after (OPN+) exogenous OPN treatment. The organoid numbers were normalized to nontreated control in each group. **f** Representative images of gastric organoids under various treatments as shown. DAPI counterstaining (**a**). Scale bar in **f** represents 25 μm (**a**, top panels), 12.5 μm (**a**, bottom panels), and 500 μm (**f**). *P < 0.05, **P < 0.01, ***P < 0.001, two-tailed unpaired *t*-tests. All error bars denote s.d. Source data are provided as a Source Data file.

CD44 contributes to the regulation of stemness, cell proliferation, and differentiation in stem/progenitor cells and cancer-propagating cells^37^. To test the role of CD44 in the regeneration and malignant transformation of the epithelium in gastric SCJ and antral regions, we reduced *Cd44* gene expression using CRISPR/Cas9-mediated genome editing, which targeted *Cd44* promoter, in the gastric organoids derived from *Lgr5*^*eGFP*−*Ires*−*CreERT2*^*Trp53*^*loxP/loxP*^*Rb1*^*loxP/loxP*^Ai9 mice (Fig. 4a). Reduction of CD44 expression resulted in decreased formation frequency and size of organoids in both gastric regions (Fig. 5b–d and Supplementary Fig 9). In SCJ epithelium, adenovirus Cre-mediated (Ad-Cre) inactivation of floxed *Trp53* and *Rb1* genes led to increase in the frequency and size of organoids and their changes to dysplastic morphology (Fig. 4b–d), similar to our experiments with CRISPR/Cas9-mediated *Trp53* and *Rb1* inactivation. These effects were abrogated by reduction of *Cd44* expression indicating critical role of CD44 in the transformation. At the same time, deletion of *Trp53* and *Rb1* was unable to promote formation of antral organoids, consistent with our observation of lower frequency of neoplastic lesions in the antral region (Supplementary Fig 9).

### OPN promotes CD44-mediated effects on SCJ epithelium

CD44 ligand, OPN, is a secreted, sialic acid-rich, glycosylated phosphoprotein encoded by *Spp1* gene^38^. Consistent with CD44 functions, OPN is implicated in tissue remodeling, wound healing, regeneration, angiogenesis, and carcinogenesis^38,39^. According to immunofluorescence staining, the first pit of the gastric SCJ contained a large fraction of OPN^+^ cells. These cells were mainly present at the lower half of the pit (Fig. 5a, b) and co-expressed CD44 (97.5%). Few OPN^+^ cells (2.7%) co-localized with the Lgr5^+^ stem cells at the bottom of the pit (Fig. 5c). Majority of OPN^+^ cells also co-localized with CD44 in the antral region. They were present only in the immediate vicinity of Lgr5^+^ stem cell niche and were far smaller in number as compared with SJC pit (Fig. 5a–c).

To investigate the functional role of OPN in gastric epithelial homeostasis and carcinogenesis, we exposed primary organoid cultures of SCJ and antral regions prepared from mice with floxed *Trp53* and *Rb1* to exogenous OPN. SCJ but not antral organoids increased their formation after OPN addition. This effect was similar in the wild-type (Ad-*Cre*^*−*^) and *Trp53/Rb1-*deficient (Ad-*Cre*^*+*^) SCJ organoids (Fig. 5d–f). Consistent with the key role of CD44 in OPN signaling, CRISPR/Cas9-mediated reduction of CD44 expression abolished OPN-induced increase in organoid formation (Fig. 5e, f).

**Fig. 6:**
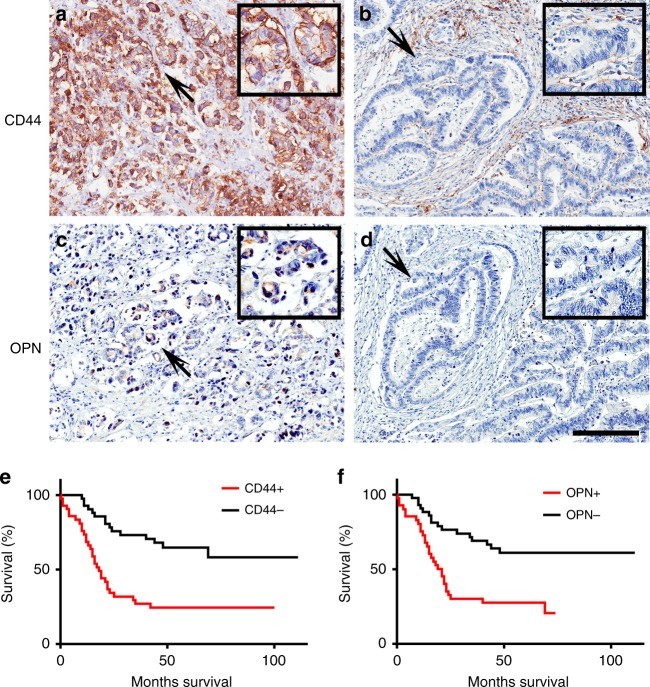
CD44 and OPN expression in human GEJ cancer correlates with the worse survival of patients. Representative images of cancer cells with (**a**, **c**) and without CD44 (**a**, **b**) and OPN **(c**, **d**) expression. Arrows indicate areas shown in insets. **e**, **f** Kaplan–Meier survival analysis of GEJ cancer patients with CD44 (**e**, CD44 + *n* = 42, CD44− *n* = 42, *P* < 0.0001) and OPN (**f**, OPN+ *n* = 41, OPN− *n* = 43, *P* = 0.0002) expression. Combined data set. Elite ABC method and hematoxylin counterstaining (**a**–**d**). Scale bar in **d** represents 200 μm (**a**–**d**) and 100 μm (insets in **a**–**d**). Source data are provided as a Source Data file.

To determine the clinical relevance of the CD44 and OPN expression in human GEJ cancer, we immunostained both proteins in two patient cohorts. Expression of both proteins highly correlated with shorter postoperative survival time of patients (Fig. 6 and Supplementary Fig. 10). In a combined dataset, positive immunoreaction with CD44 and OPN was observed in 50% (42/84) and 48.8% (41/84) of cases, respectively.

## Discussion

**Fig. 7:**
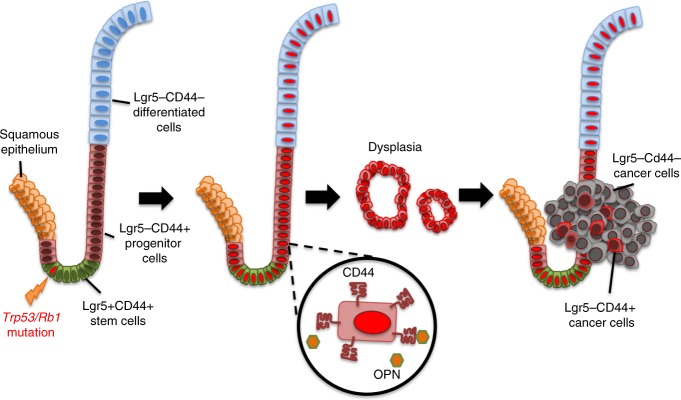
Carcinogenesis at the gastric squamous-columnar junction.

We introduce a model of metastatic gastric SCJ carcinoma, show that this malignancy likely arises from actively proliferating immature Lgr5^−^CD44^+^ progeny of Lgr5 cells, and identify CD44–OPN pathway as a critical mechanism for SCJ carcinogenesis (Fig. 7). The first pit of gastric glandular epithelium has a unique anatomic structure with distinct cellular composition. Resembling the antral rather than the corpus glands it lacks secretory parietal cells^40^, but contains Lgr5^+^ cells participating in the long-term physiological homeostasis according to our cell lineage tracing studies and previous publications^33,34^. Thus, it is well suitable for comparing cancer susceptibility in two distinct gastric regions.

Previously, it has been suggested that the preferential transformation of the first pit may be attributable to the retention of a distinct embryonic epithelial lineage at the gastric SCJ^19^. These unique embryonic epithelial cells express both cytokeratin 7 and MUC4, cover the entire luminal surface of the stomach during embryogenesis, and persistently remain at the gastric SCJ junction of adult mice and humans^19^. Upon programmed injury via diphtheria toxin A expression in the cytokeratin 14-positive squamous epithelium, these residual embryonic cells shift toward the squamous forestomach and recapitulate early Barrett’s-like epithelium at the junction region^19^.

It has also been reported that the Barrett’s-like metaplasia induced by overexpression of cytokine IL-1 may develop from the proximal expansion of Lgr5^+^ stem cell progeny^11^. Our mouse model and organoid assays further support the paradigm that at least a part of gastroesophageal carcinomas arise from the gastric glandular epithelium, as opposed to the squamous epithelium. At the same time, in our mouse model we have not found classical Barrett’s esophagus-like lesions. Thus it may represent a new system to study a group of gastric SCJ cancers, which do not progress through Barrett’s esophagus stage. Since mouse SCJ is similar but not identical to human gastroesophageal junction, further studies based on human tissues, such as organoids, should facilitate testing this possibility.

Previously it has been shown that deletion of *Apc* in Lgr5^+^ cells leads to formation of gastric antral adenomas^33^. It has also been reported that inactivation of *Smad4* and *Pten* in Lgr5^+^ cells leads to invasive intestinal type carcinomas in antral and SCJ regions at 40% and 25% frequency, respectively^41^. In both models, mice developed extensive intestinal tumors, thereby preventing long-term studies of gastric neoplastic progression, including metastasis. These models also highlight the importance of initiating genetic alterations necessary for the accurate modeling of a particular type of gastric cancer. As our study shows, inactivation of *Trp53* and *Rb1*, which are a part of commonly affected pathways in human gastroesophageal cancer, leads to preferential formation of SCJ carcinomas arising with 100% penetrance. Importantly, these carcinomas are similar to human gastroesophageal carcinomas by their genetic makeup and clinical features. Thus, the mouse model of metastatic gastric carcinoma established by us should be useful for further studies of carcinomas of the proximal stomach. It may also provide further insights into the mechanisms responsible for cancer-prone properties of epithelial TZs.

The role of Lgr5 signaling in cancer development and metastasis remains controversial^42^. Lgr5 has been recognized as a stem cell marker expressed by the proliferating adult stem cells in the intestine. Such cells possibly represent the cells of origin of some intestinal neoplasms^43,44^. Being an enhancer of Wnt/β-catenin signaling pathway, Lgr5 is involved in oncogenic activities of this pathway, such as cell proliferation, migration, colony formation, and tumorigenicity in a wide variety of cancers^34,44–49^. Conversely, the tumor-suppressive function of Lgr5 has also been described on the basis of several clinical and experimental studies. Epigenetic methylation of *Lgr5* is commonly observed in the colon cancer patients, who have higher tumor grades and poor prognosis^50^. Reexpression of Lgr5 allows cancer cells to decrease the clonogenicity and tumorigenicity^50^. Through interaction with its ligand R-spondin 2, Lgr5 functions as a negative regulator of Wnt-signaling to suppress the cancer proliferation and metastasis^51^. Lgr5 has also shown to be directly involved in the activation of TGF-β signaling and suppression of colon cancer metastasis^52^.

Our results suggest that Lgr5 signaling is not an essential driver of some cancers arising after introduction of cancer-initiating genetic alterations into Lgr5^+^ stem cells. In our study Lgr5^−^CD44^+^ cells of the first gastric gland constitute the majority of early dysplastic lesions, and no Lgr5^+^ neoplastic cells are observed during SCJ carcinogenesis. These results are consistent with our organoid culture experiments, where we show that Lgr5^−^CD44^+^ cells, but not Lgr5^+^CD44^+^ or Lgr5^−^CD44^−^ cells, can be easily transformed by inactivation of *Trp53* and *Rb1*. These findings point to Lgr5^−^CD44^+^ cells as the most likely cell of gastric SCJ carcinoma origin. It remains to be investigated if Lgr5 signaling mechanisms play tumor-suppressive role in SCJ glandular stem cells, or Lgr5^+^ cells may be outcompeted by rapidly expanding Lgr5^−^CD44^+^ cells.

Our findings indicate that presence of expanded pools of immature cells may explain the susceptibility of gastric SCJ to the malignant transformation. Many recent studies have experimentally induced carcinogenesis in various organs by selectively introducing oncogenic alterations into adult stem cells^53–55^. In actively renewing tissues, like gastrointestinal epithelium, proliferating stem cells will rapidly transmit their mutational load to their progeny during the routine renewing process. It is possible that due to changes in microenvironment or intrinsic mechanisms the progeny of such mutant cells is more susceptible to the malignant transformation. It also has been previously reported that the differentiated progeny may acquire the capacity to dedifferentiate to stem-like state during the carcinogenesis^56,57^. In such scenario, immature status of cells may provide optimal conditions for acquiring cancer-prone status. In vivo experiments using specific targeting of stem cells vs their immature progeny should directly test such possibilities. It may be of interest to investigate if other cancer-prone epithelial TZs also have large pools of immature cells.

Our study identified OPN–CD44 signaling as a key mechanism in the transformation of gastric SCJ glandular epithelium. OPN–CD44 signaling is known to regulate the stem/progenitor cell proliferation and differentiation in liver and hematopoietic systems, and also to promote stemness of melanoma, glioma, and colon cancers^38,58–60^. It has been reported, that through binding to cell surface receptor CD44, perivascular niche-derived OPN promotes the stemness and tumorigenicity of cancer cells via cleavage and translocation of the C-terminal intracellular domain (ICD) of CD44 into the nucleus^38^. The CD44ICD functions as a critical transcriptional factor to induce the stem-like properties in targeted cells by enhancing the hypoxic inducible factor-2α in a CBP/p300-dependent manner^61,62^. Moreover, OPN–CD44 interactions have been shown to activate downstream signaling pathways, such as phosphatidylinositol 3-kinase (PI3K)/Akt cascade^63^ and TIAM1–Rac1 signaling^64^, that are highly associated with cancer progression and metastasis via enhancing cell proliferation, survival, and mobility. Our studies did not show preferential presence of ICD in glandular cells of SCJ (Fu and Nikitin, unpublished observations). However, we have observed that the CD44^+^ immature progenitor cells at the first pit of SCJ can produce OPN. In this context, OPN may function as an essential autocrine factor responsible for increasing the stemness phenotype, thereby promoting gastric epithelial regeneration, but also facilitating malignant transformation. Further investigations are needed to identify downstream mechanisms by which OPN–CD44 signaling may selectively affect immature CD44^+^ cells in the SCJ but not antrum.

Expression of both CD44 and OPN has been reported to correlate with the worse prognosis for human gastric cancer patients^65,66^. According to TCGA data analysis, upregulation of CD44 and OPN shows a trend for the worst prognosis in esophageal carcinomas and CIN subtype of gastric carcinomas, which are predominantly located near human gastroesophageal junction^29^ Furthermore, in both cases disease/progression-free survival of patients is significantly shorter. Our studies of gastroesophageal cancers are in agreement with those observations. Thus targeting OPN–CD44 pathway may be a promising approach for diagnosis and treatment of gastroesophageal carcinoma.

## Methods

### Experimental animals

The *Lgr5*^*tm1(cre/ERT2)Cle*^*/J (Lgr5*^*eGFP*−*Ires*−*CreERT2*^) knock in mice (Stock number 008875), *Gt(ROSA)26Sor*^*tm9(CAG*−*tdTomato)Hze*^ (*Rosa-loxP-stop-loxP-*tdTomato/Ai9) mice (Stock number 007909) and NOD.Cg-*Prkdc*^*scid*^ *Il2rg*^*tm1wjl*^/SzJ (NSG) mice (Stock number 005557 were obtained from The Jackson Laboratory (Bar Harbor, ME, USA). The *Trp53*^*loxP/loxP*^ and *Rb1*^*loxP/loxP*^ mice, which have *Trp53* and *Rb1* genes flanked by *loxP* alleles, respectively, were a gift from Dr Anton Berns. *Lgr5-*DTR mice^67^ were a gift from Dr Frederic J. de Sauvage. All the experiments and maintenance of the mice were following ethical regulations for animal testing and research. They were approved by the Cornell University Institutional Laboratory Animal Use and Care Committee.

### Tamoxifen induction

For lineage tracing experiments, 6-week-old *Lgr5*^*eGFP*−*Ires*−*CreERT2*^Ai9 mice received a single dose (8 μl g^−1^ body weight) of tamoxifen (25 mg ml^−1^ in corn oil, Sigma-Aldrich, St. Louis, MO, USA, T5648) by intraperitoneal injection. At 1, 4, 10, and 200 days after induction, the tamoxifen-pulsed mice were euthanized by CO_2_ and further analyses were carried out. For tumor induction experiments, 6–10-week-old *Lgr5*^*eGFP*−*Ires*−*CreERT2*^*Trp53*^*loxP/loxP*^ *Rb1*^*loxP/loxP*^Ai9 mice and control mice were intraperitoneally injected with tamoxifen (25 mg ml^−1^ in corn oil, 8 μl g^−1^ body weight) three times, every other day for a total of 6 days to maximize the Cre-recombination efficiency. The day after first injection was counted as the first day post induction (p.i.).

### Histology, immunohistochemistry, and image analysis

All tissues were fixed in buffered 4% paraformaldehyde overnight at 4 °C followed by standard tissue processing and paraffin embedding. Histology and immunohistochemistry stainings were carried out on 4-μm-thick tissue sections. For immunohistochemistry, antigen retrieval was performed by incubation of deparaffinized and rehydrated tissue sections in boiling 10 mM sodium citrate buffer (pH 6.0) for 10 min. The primary antibodies against GFP (NOVUS biological, Littleton, CO, USA; NB600-303; 1:8000), CD44 (Santa Cruz Biotechnologies, Dallas, TX, USA; sc-18849; 1:1000), Mucin5AC (Abcam, Cambridge, UK; ab3649; 1:500), H^+^K^+^-ATPase (MBL international corp., Woburn, MA, USA; D032-3H; 1:500), chromogranin A (Santa Cruz, sc-1488; 1:2000), pepsinogen C (Abbexa, Cambridge, UK; abx002093; 1:400), OPN (R&D Systems, Minneapolis, MN, USA; AF808; 1:200 and Sigma, HPA027541; 1:200), RFP (Rockland Immunochemicals Inc., Pottstown, PA, USA; 600-401-379S; 1:400),Ki67 (Abcam, ab16667; 1:400), and KRT5 (BioLegend, San Diego, CA, USA, PRB-160P; 1:400) were incubated at room temperature (RT) for 1 h, followed by incubation with secondary biotinylated antibodies (30 min, RT). Modified Elite avidin–biotin peroxidase (ABC) technique (Vector Laboratories, Burlingame, CA, USA; pk-6100) was performed at RT for 30 min. Hematoxylin was used as the counterstain. For immunofluorescence, the primary antibodies against GFP (NOVUS biological; NB600-303; 1:1000), CD44 (Santa Cruz Biotechnologies; sc-18849; 1:200), OPN (R&D Systems; AF808; 1:100), RFP (Rockland Immunochemicals Inc; 600-401-379S; 1:100) were incubated at RT for 1 h, followed by incubation with secondary antibody conjugated with indicated fluorescence protein for 30 min at RT. DAPI (Sigma; 32670-5MG-F, 1:200) were used for nuclear counterstaining. All antibodies used for immunostaining are listed in Supplementary Table 2. For quantitative studies, sections were scanned by ScanScope CS2 or FL (Leica Biosystems, Vista, CA) with a 40× objective, followed by the analysis with the ImageJ software (National Institutes of Health, Bethesda, MD, USA). Carcinomas were considered to be positive for CD44 and OPN1 in cases with over 10% stained neoplastic cells.

### BrdU incorporation assay

A single dose (20 μl g^−1^ body weight) of BrdU (2.5 mg ml^−1^, Sigma; B5002) was administered to mice by intraperitoneal injection. Mice were euthanized using CO_2_ 2 h after BrdU injection, and the stomachs were collected and fixed with 4% paraformaldehyde overnight at 4 °C. Deparaffinized tissue sections were exposed to 10-min boiling in 10 mM citrate buffer followed by incubation with 4 N hydrogen chloride (HCl, RT, 10 min). The anti-BrdU primary antibody (Abcam; ab2284;1:100) was incubated at RT for 1 h, followed by Alexa 594 conjugated secondary antibody (Invitrogen, Carlsbad, CA, USA; A11016; 1:200) for 30 min at RT. DAPI (Sigma; 32670-5MG-F) were used for nuclear counterstaining.

### Isolation of primary gastric epithelial cells

Individual stomachs were isolated from 6 to 10 week-old *Lgr5*^*eGFP*−*Ires*−*CreERT2*^*Trp53*^*loxP/loxP*^*Rb1*^*loxP/loxP*^Ai9, *Trp53*^*loxP/loxP*^*Rb1*^*loxP/loxP*^Ai9 or *Lgr5*-DTR mice, washed with ice-cold PBS buffer several times, dissected into small pieces (<2 mm in length), and incubated in gentle dissociation buffer (Stem cell technologies, Vancouver, Canada; 07174) at RT for 15 min. After removal of the dissociation buffer, the tissue fragments were vigorously pipetted for 10–20 times with ice-cold 0.1% BSA/PBS solution using 10 ml pipette, and transferred to 50 ml conical tube. The gastric epithelial cells were collected and counted after centrifugation and resuspension using DMEM F12 50/50 medium (Corning Inc., Corning, NY, USA; 10-092-CV).

### Gastric organoid culture

The primary gastric organoid culture was established by modifying previously described methods^68^. Primary mouse gastric epithelial cells (10^4^) were suspended in 0.1 ml of DMEM F12 50/50 medium, and 1:1 mixture with liquid growth factor-reduced Matrigel (Corning, 354230), and plated around the rim of the well of a 24-well tissue culture plate. Matrigel was allowed to solidify in the 37 °C incubator for 20 min, and overlaid by stem cell culture medium (IntestiCult^TM^ Organoid Growth Medium [Stemcell Technologies; 6005] supplemented with 10 nM gastrin [Sigma; G9020]) in the first 3 days. Each well was washed by PBS twice following by addition of differentiation medium (Advanced DMEM/F12 50/50 medium supplemented with 1 μg ml^−1^ R-spondin1 [PeproTech Inc., Rocky Hill, NJ, USA; 120-38] and 50 ng ml^−1^ EGF [PeproTech, 315-09]) during subsequent 4 days. For culturing Lgr5^+^ organoids, small molecules CHIR99021 (2 μM, Stemcell Technologies, 72052) and valproic acid (2 mM, Stemcell Technologies, 72292) were added to stem cell culture medium. For culturing Lgr5^−^CD44^+^ organoids, diphtheria toxin (2 ng ml^−1^, Sigma, D0564) was added to stem cell culture medium to abolish the Lgr5^+^ cells. For culture of Lgr5^−^CD44^−^ organoids, diphtheria toxin (2 ng ml^−1^, Sigma; D0564) was also added to differentiation medium as described above.

For passaging, 1 ml of Accumax cell dissociation solution (Innovative Cell Technologies, San Diego, CA, USA; AM105-500), was added to each well followed by incubation at 37 °C for 10 min. The organoids were separated into single cells upon vigorous pipetting, and transferred into 15 ml conical tube. After double washing with 10 ml DMEM/F12 50/50 medium, the cells were counted and cultured as described above. In experiments with adenovirus-mediated Cre (AdCre) recombination, organoids were treated with Cre-expressing adenovirus (2 × 10^7^ pfu in stem cell culture medium) at 37 °C for 2 h on the second day of culture. Blank adenovirus was used as control under same conditions. In the lentiviral CRISPR/Cas9-mediated experiments, the recombinant lentivirus was added to the stem cell culture medium during first 3 days after plating.

### Quantitative reverse transcription real-time PCR (qRT-PCR)

RNA from organoids was isolated using mirVana miRNA Isolation Kit (Thermo Fischer Scientific; AM1560). cDNA synthesis was performed using SuperScript III First Strand system (Invitrogen; 18080-400) in accordance with manufacturer’s protocol. Quantitative RT-PCR was carried out with SYBR Green (Quanta BioSciences, Beverly, MA, USA; 95054-100) according to manufacturer’s instruction. *Lgr5* expression was assessed using forward primer (TCTTCTAGGAAGCAGAGGCG) and reverse primer (CAACCTCAGCGTCTTCACCT) and its relative expression was normalized to β-actin expression with a use of forward primer (GATTACTGCTCTGGCTCCTAGC) and reverse primer (GACTCATCGTACTCCTGCTTGC).

### Construction of CRISPR plasmids

The lentiCRISPR v2 was obtained from Addgene (Cambridge, MA, US; 52961). The *Cd44-*sgRNA (CRISPR-*Cd44*) sequences (CTGGAGAACGTGGGCGCACG) were designed using web tools Optimized CRISPR Design (http://crispr.mit.edu) and CHOPCHOP (https://chopchop.rc.fas.harvard.edu). Insertion of sgRNA was performed following previously published protocol^69^. The lentiCRISPR v2 plasmid was digested by FastDigest *BsmBI* (Thermo Fischer Scientific; FD0454). The sgRNA dimers were phosphorylated by T4 polynucleotide Kinase (NEB) and ligated into plasmid using Rapid DNA Ligation Kit (Thermo Fischer Scientific; K1423s). For CRISPR-*Trp53* and CRISPR-*Rb1* experiments three sets of *Trp53-*sgRNAs (CRISPR-*Trp53a:* AGTGAAGCCCTCCGAGTGTC, CRISPR-*Trp53b:* GAAGTCACAGCACATGACGG, CRISPR-*Trp53c:* AAATTTGTATCCCGAGTATC) and three sets of *Rb1-*sgRNAs (CRISPR-*Rb1a:* TGTAGCTCAGTAAAAGTGAA, CRISPR-*Rb1b:* TTGGGAGAAAGTTTCATCCG, CRISPR-*Rb1c:* AGAAATCGATACCAGTACCA) were separately inserted into the lentiCRISPR v2 plasmid following manufacture’s recommendation. Inactivation of genes was confirmed by QRT-PCR detection of gene expression and Sanger sequencing (Supplementary Fig. 11). To analyze the frequency of *Cd44* mutations DNA isolated from individual cells infected with CRISPR-Cd44 was extracted using the Nucleospin Tissue XS kit (TakaBio). The *Cd44* locus was amplified using primers CD44_F: GatccgCTCGAGactgagaggggcgaggtctt and CD44_R: CGCgatcACTAGTagcgagggggctgtgactaa and NebNext Q5 High Fidelity polymerase. The obtained DNA samples were purified using QIAquick PCR purification kit and sequenced using the CD44 (F) primers. The obtained raw data was then manually assessed.

### OPN treatment

The organoids were passaged into 24-well plate 1 day prior treatments. On the next day, the old medium was removed and replaced by fresh Stem Cell Culture Medium with recombinant OPN (1 μg ml^−1^; Sigma; SRP3131). The medium was changed and added with each factor every other day.

### RNA sequencing

SCJ tumors (*n* = 5) were dissected from stomachs of *Lgr5*^*eGFP*−*Ires*−*CreERT2*^*Trp53*^*loxP/loxP*^*Rb1*^*loxP/loxP*^Ai9 mice at least 250 days after tamoxifen induction. The tumor borders were delineated based on tdTomato expression. The wild-type control stomachs (*n* = 3) were isolated from age-paired mice and separated into squamous forestomach, SCJ and antral compartments. All the tumors and gastric tissue were minced on ice using preautoclaved glass tissue grinder. The total RNA was purified from digested tissue using mirVana miRNA Isolation Kit (Thermo Fischer Scientific; AM1560). Following quality assessment by Agilent BioAnalyzer, samples with RNA Quality Number (RQN) greater than 7.0 were submitted to the Cornell RNA Sequencing Core (RSC) to generate standard library using the next generation high throughput sequencing by Illumina TruSeq system (Illumina, San Diego, CA, USA).

For RNAseq transcription data cutadapt v1.8 was used to trim and filter reads and cuffquant was used to quantify transcripts for annotated genes (UCSC mm10). For gene expression data set the FPKM values were generated with cuffnorm v2.2.1. GSEA implemented with GSEA v2.2.3 software (http://software.broadinstitute.org/gsea/index.jsp) were performed through the use of previously defined human gastroesophageal junction cancer signature genes^70^ as the gene set and the preranked 23,361 genes defined above as the expression data set. In addition, the Broad Institute’s Hallmark, Oncogenic Signature, Motiff, and Curated Signature gene sets were used for the analysis. Further canonical pathway analysis was performed using GIAGEN’s Ingenuity Pathway Analysis (IPA) software (https://www.qiagenbioinformatics.com).

### Transplantation

Dissociated cells were collected by centrifugation at 500 *g* for 5 min, resuspended with 50% of complete culture medium and 50% of high concentration Matrigel (Corning; 354263) in total volume of 100 μl, and subcutaneously injected (5 × 10^5^ cells or as indicated) in the flanks of NSG mice.

### Patient materials

Two patient cohorts were used in this study. In first cohort, paraffin-embedded human gastroesophageal tumor specimens were obtained from tissue bank of Department of Pathology and Key Laboratories for Xinjiang Endemic and Ethnic Diseases, Shihezi University School of Medicine. Clinical characteristics of the patients are listed in Supplementary Table 2. The study obtained informed consent from all participants. The study protocol with all relevant ethical regulations for work with human participants was approved by the Ethics Committee of the First Affiliated Hospital, Shihezi University School of Medicine. For the second cohort, tissue microarray of gastroesophageal junctional carcinoma and matched normal adjacent tissue, with survival data, was obtained from US Biomax (Derwood, MD; HGEj-Ade130Sur-01). These specimens represent de-identified archive specimens collected by provider not involved in our research. As such, they do not meet definitions of “human participant research” under US federal regulations and Cornell IRB rules.

### Statistical analyses

Statistical comparisons were performed using a two-tailed unpaired *t* test and a chi square test with InStat 3 and Prism 6 software (GraphPad Software Inc., La Jolla, CA, USA). Survival curves were computed using the Kaplan–Meier method and the survival comparisons were analyzed by log-rank tests. Significance was determined as *P* < 0.05.

## Supplementary information

Supplementary Information

## Data Availability

The RNA-seq data reported in this paper is deposited in Gene Expression Omnibus (GEO); accession number: GSE130003. The Source Data provide data for all results requiring quantification. Any additional data supporting the findings of this study are available from the corresponding author upon reasonable request.

## Source data

Source Data
