# Supplementary material for: Gastric squamous-columnar junction contains a large pool of cancer-prone immature osteopontin responsive Lgr5−CD44+ cells
Source: Nat Commun. 2020 Jan 3;11:84. doi: 10.1038/s41467-019-13847-2 (PMC6941991; doi:10.1038/s41467-019-13847-2)
Supplement: Supplementary file 1 — Supplementary Information [file 41467_2019_13847_MOESM1_ESM.pdf]

Supplementary Information

Gastric squamous-columnar junction contains a large pool of cancer-prone immature osteopontin responsive $Lgr5^{-}CD44^{+}$ cells

Fu et al.

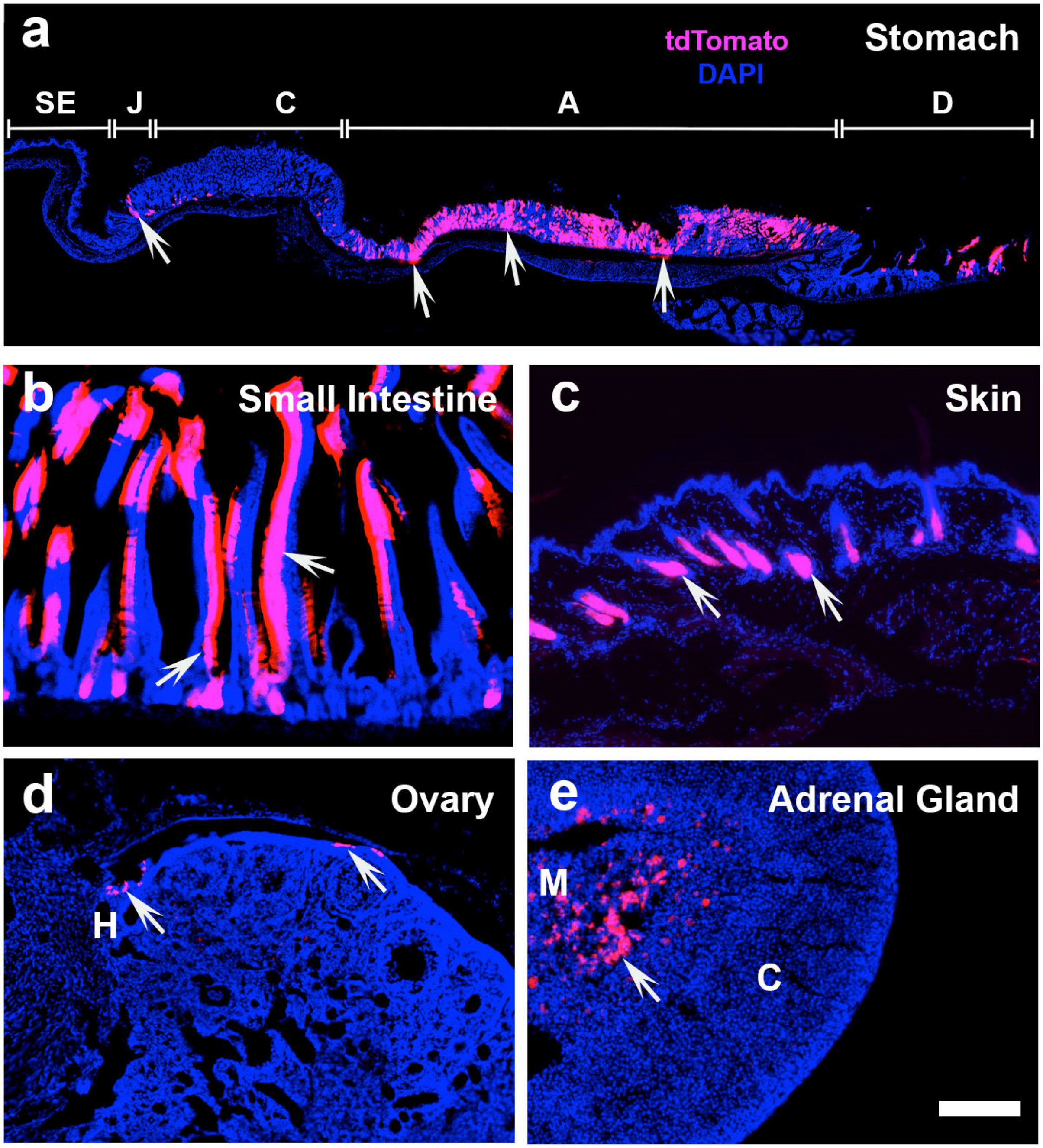

Supplementary Figure 1. Detection of Lgr5⁺ stem cell lineages in multiple organs. **a-e**, Expression of tdTomato (magenta) in organs of *Lgr5$^{eGFP\text{-}Ires\text{-}CreERT2}$*Ai9 mice 200 days post induction with tamoxifen. Positive cells (arrows) are present at the squamous-columnar junction (SCJ) and antrum of stomach (**a**), and in the small intestine (**b**), hair follicles (**c**), ovarian surface epithelium (**d**), and adrenal gland medulla (**e**). SE, squamous epithelium; J, SCJ; C, corpus; A, antrum; D, duodenum; H, hilum; M, medulla; C, cortex. Counterstaining with DAPI (blue). Scale bar in **e** represents 1 mm (**a**), 300 μm (**b**, **c** and **e**) and 230 μm (**d**).

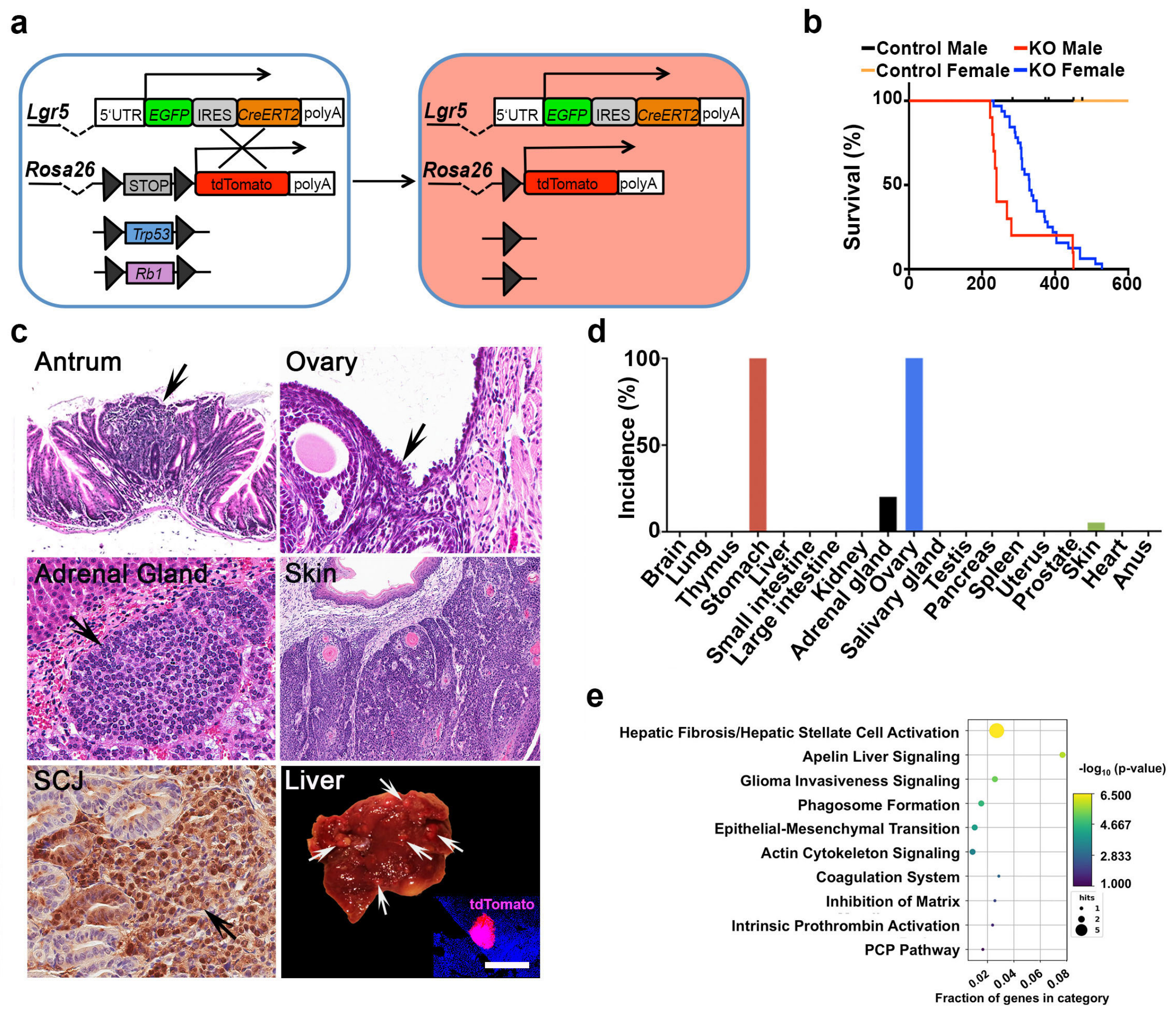

Supplementary Figure 2. Inactivation of *Trp53* and *Rb1* genes in Lgr5⁺ stem cell using *Lgr5*$^{eGFP\text{-}Ires\text{-}CreERT2}$*Trp53*$^{loxP/loxP}$*Rb1*$^{loxP/loxP}$Ai9 mice. a, Experimental strategy for inactivation of *Trp53* and *Rb1* in Lgr5^{+} stem cells. **b**, Male mice with conditional knockout (KO) of *Trp53* and *Rb1* have significantly shorter survival span (median=239 days, n=10) compared to female mice with the same KO (median=329 days, n=32). $P<0.05$. Male (n=5) and female (n=4) *Lgr5*$^{eGFP\text{-}Ires\text{-}CreERT2}$*Trp53*$^{loxP/loxP}$*Rb1*$^{loxP/loxP}$Ai9 mice without tamoxifen induction are used as controls. Kaplan-Meier survival curve. **c,** Primary and metastatic neoplasms in *Lgr5*$^{eGFP\text{-}Ires\text{-}CreERT2}$*Trp53*$^{loxP/loxP}$*Rb1*$^{loxP/loxP}$Ai9 mice. Antrum**,** antral adenoma (arrow). 309 days post induction (p.i.) with tamoxifen. Ovary, dysplasia (arrow) of the ovarian surface epithelium in the ovarian hilum region. 253 days p.i. Adrenal gland, pheochromocytoma (arrow) in the adrenal gland medulla. 231 days p.i. Skin, squamous cell carcinoma of the skin. 235 days p.i. expression of tdTomato in primary gastric SCJ neoplasm (arrow), 222 days p.i. Liver, gross and microscopic (inset) image of liver metastases (arrows). tdTomato, expression of tdTomato (magenta) in metastatic node (arrow). Staining with hematoxylin and eosin (Antrum-Liver), and counterstaining with DAPI (blue, tdTomato inset). Scale bar in tdTomato image represents 250 µm (Antrum, Skin, and tdTomato inset), 60 µm (Ovary and Adrenal gland), 50 µm (SCJ), 7.3

mm (Liver), and 620 μm (tdTomato inset). **d**, Incidence of neoplastic lesions in various organs of mice with conditional KO of *Trp53* and *Rb1 (Trp53*$^{-/-}$*Rb1*$^{-/-}$; n=20). **e,** Ingenuity Pathway Analysis (IPA) of human gastroesophageal cancer signature genes which are upregulated in mouse SCJ cancer model. Source data are provided as a Source Data file.

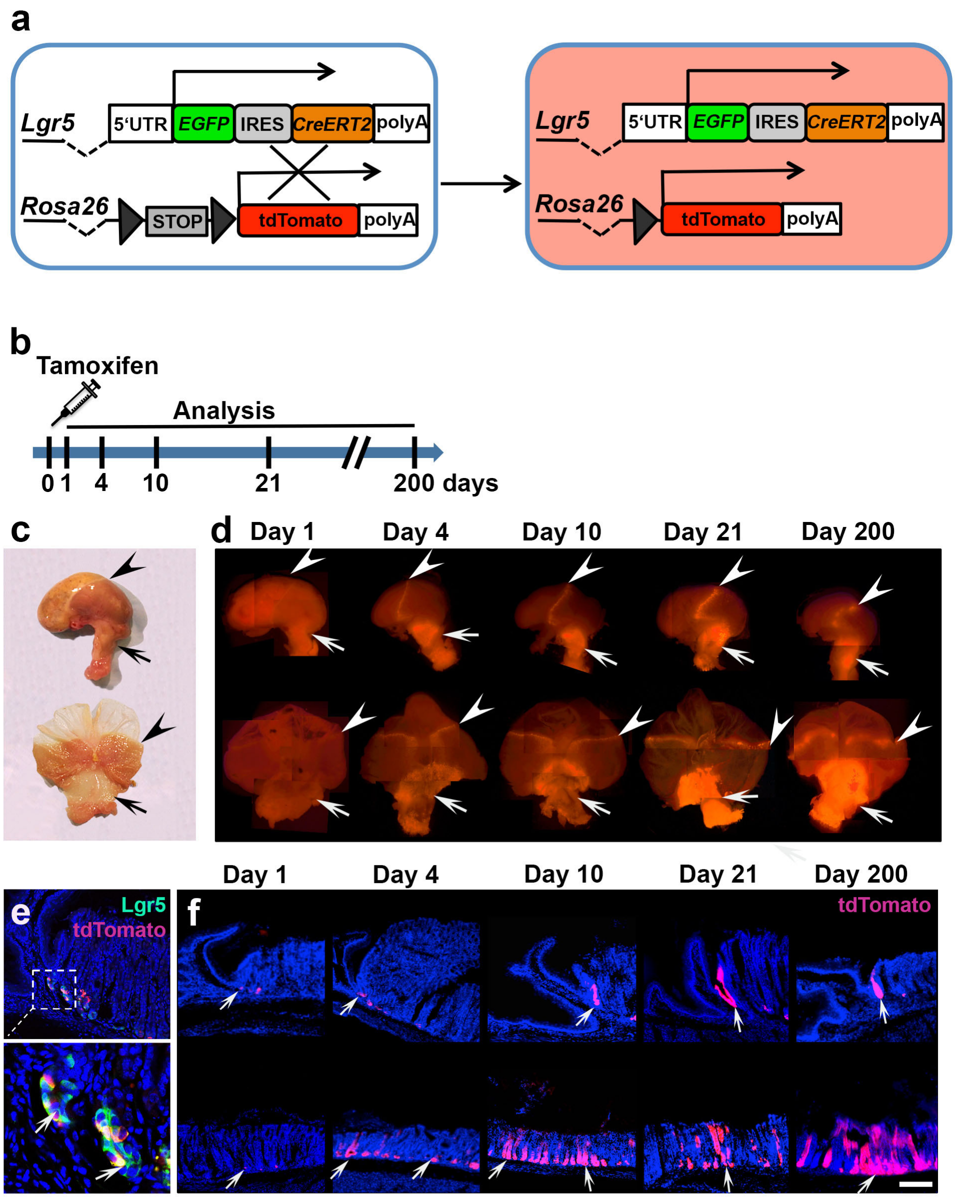

Supplementary Figure 3. Lineage tracing of Lgr5⁺ stem cells in the mouse stomach. **a**, Experimental design of Cre-mediated lineage tracing in *Lgr5*$^{eGFP\text{-}Ires\text{-}CreERT2}$Ai9 mice. **b**, Schedule of tamoxifen administration and material collection. **c** and **d**, Macroscopic light (**c**) and fluorescence (**d**) images of the intact (top) and dissected (bottom) stomachs of *Lgr5*$^{eGFP\text{-}Ires\text{-}CreERT2}$Ai9 mice collected at indicated days p.i. SCJ, arrowhead; Antrum, arrows. **e**, Detection of co-localized expression of Lgr5-eGFP (turquoise) and tdTomato (magenta) in Lgr5⁺ cells at SCJ (arrows in bottom image) one day p.i. Rectangle in top image indicates area shown in bottom image. **f**, Detection of tdTomato expression cells (magenta, arrows) in the longitudinal sections of SCJ (top) and antrum (bottom) of stomachs of *Lgr5*$^{eGFP\text{-}Ires\text{-}CreERT2}$Ai9 mice. Scale bar in **f** represents 3 mm (**c** and **d**), 100 µm (**e**, top panel), 25 µm (**e**, bottom panel), and 150 µm (**f**).

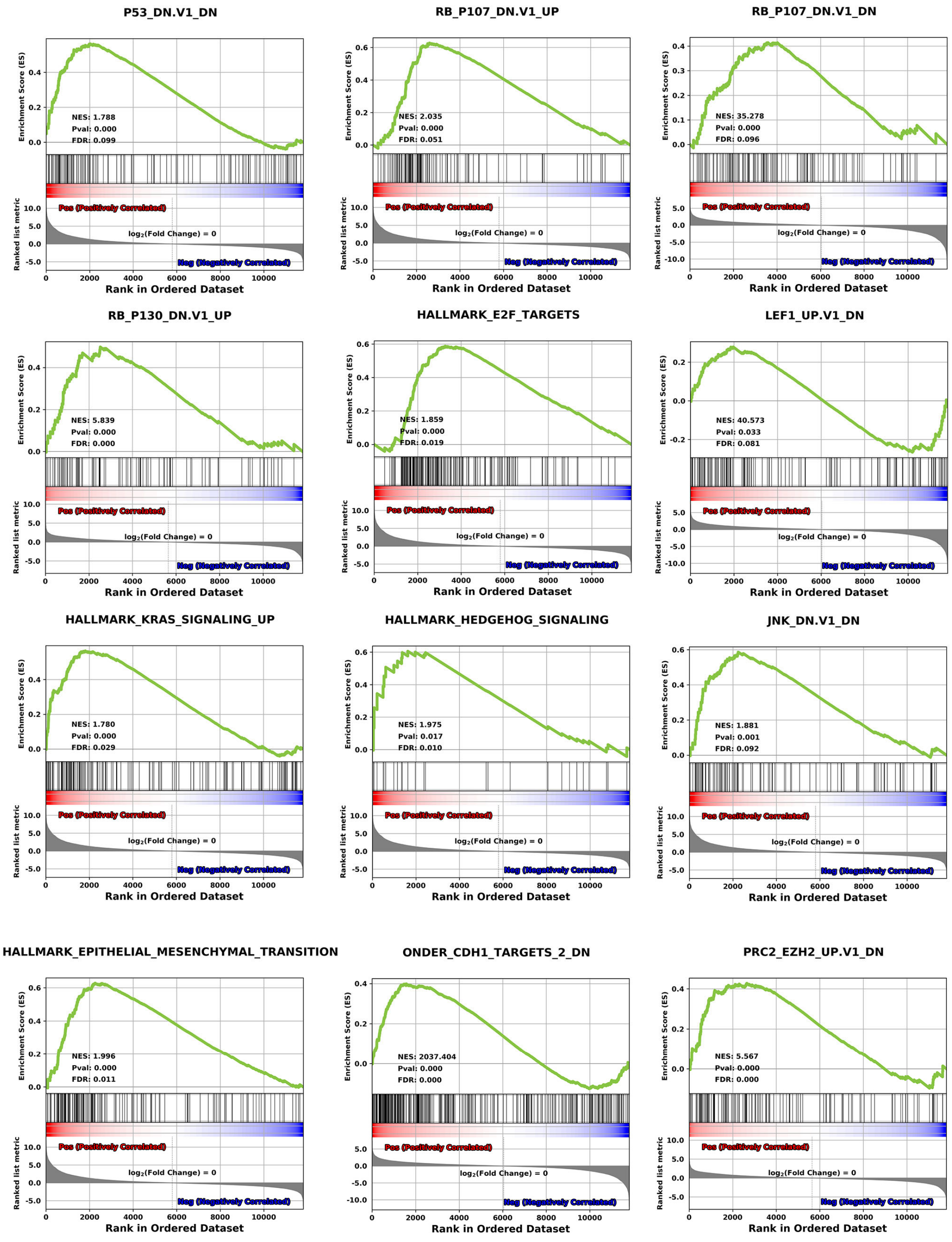

Supplementary Figure 4. Gene Set Enrichment Analysis of RNA-seq data of mouse gastric SCJ cancers using the Broad Institute's gene sets. Normalized enrichment scores (NESs), P value, and false discovery rate (FDR) are shown.

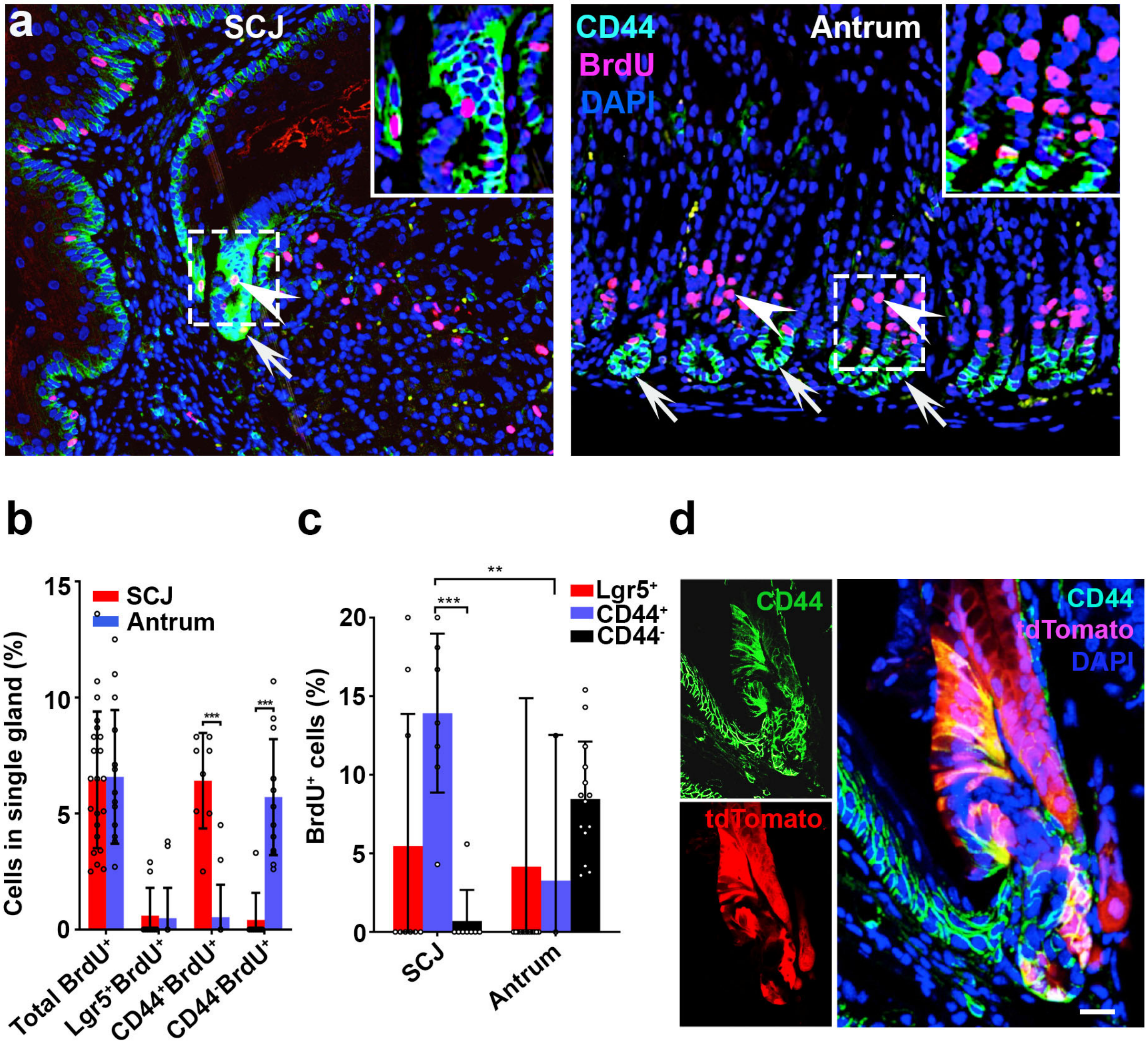

Supplementary Figure 5. Characterization of cells in the gastric SCJ and antrum. a, Detection of cells expressing CD44 (turquoise, arrows) and incorporating BrdU (magenta, arrowheads) in the SCJ (left) and antrum (right). Punctate rectangles indicate areas shown in insets. **b**, Quantification of $Lgr5^+$ and $CD44^+$ cells incorporating BrdU per gland ($n \geq 8$ in each group). **c.** Percentage of $BrdU^+$ cells in $Lgr5^+$, $CD44^+$, and $CD44^-$ fractions ($n \geq 8$ in each group). **d.** Detection of CD44 (green, left panel; turquoise, right panel), tdTomato (red, left panel; magenta, right panel) and overlay (orange) in Lgr5 progeny of SCJ epithelium of *Lgr5*$^{eGFP\text{-}Ires\text{-}CreERT2}$Ai9 mice 200 days p.i. Counterstaining with DAPI (**a**). Scale bar in **d** represents 20 µm (a and left panels of **d**) and 10 µm (insets in **a** and right panel of **d**). **P<0.01, ***P<0.001, two-tailed unpaired t-tests. All error bars denote s.d. Source data are provided as a Source Data file.

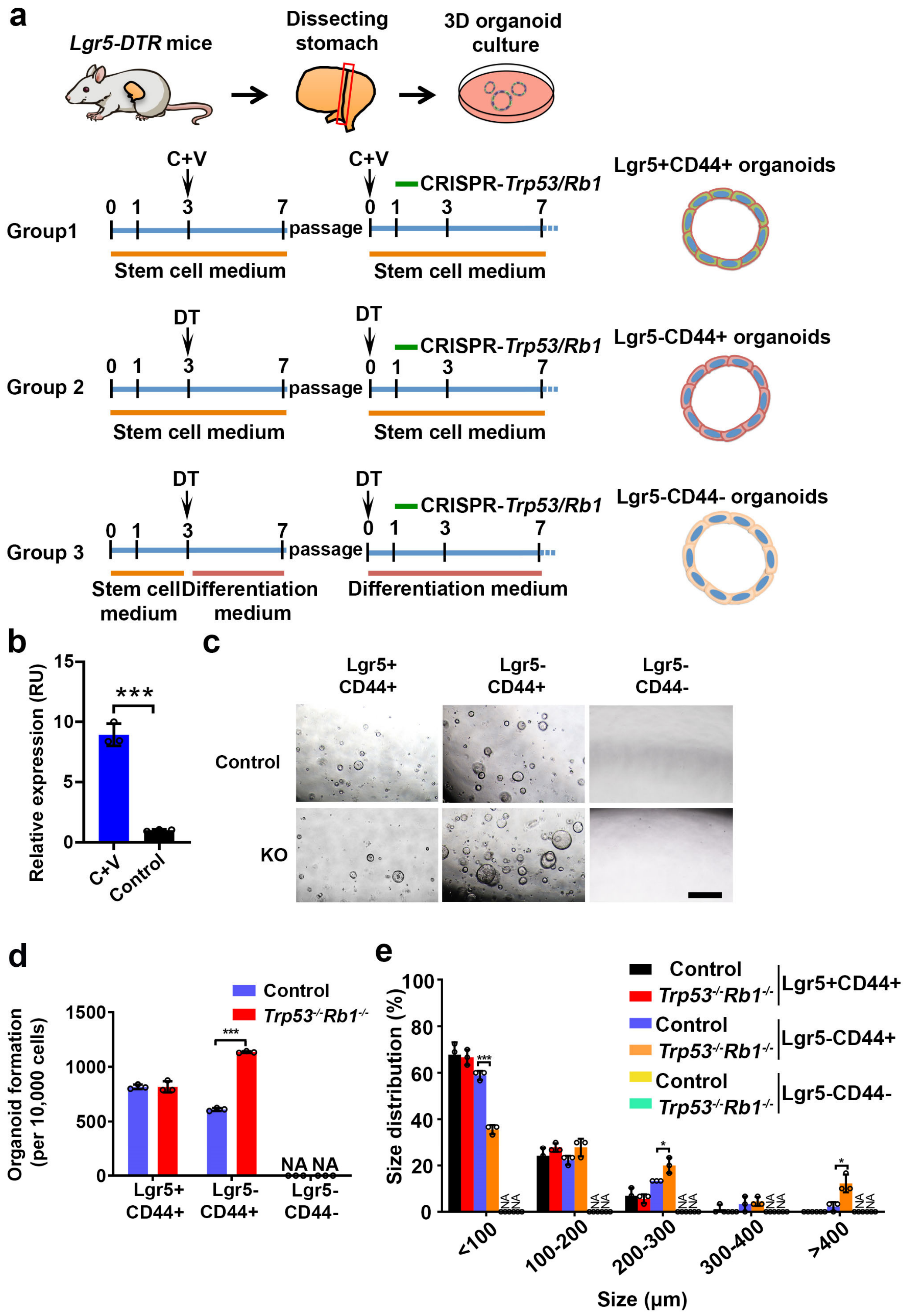

Supplementary Figure 6. The effect of *Trp53* and *Rb1* inactivation on gastric SCJ organoids maintained at different differentiation stages. a, Experimental design. The rectangle indicates region used for cell isolation. See legend to Figure 3 for further details.

b, *Lgr5* expression in organoids cultured in presence (C+V) or absence (control) of CHIR99021 (C) and valproic acid (V) (n=3 in each group). QRT-PCR. **c**, Representative phase contrast images of SCJ organoids derived from *Lgr5*-DTR mice. C, CHIR99021; V, valproic acid; DT, diphtheria toxin. Scale bar represents 500 µm. NA, not available. *P<0.05, ***P<0.001, two-tailed unpaired t-test. All error bars denote s.d. **d** and **e**, Numbers (**d)** and sizes (**e**) of SCJ organoids derived from *Lgr5*-DTR mice and maintained at different differentiation conditions before and after CRISPR-mediated *Trp53* and *Rb1* deletion (n=3 in each group). NA, not available. *P<0.05, ***P<0.001, two-tailed unpaired t-tests. All error bars denote s.d. Source data are provided as a Source Data file.

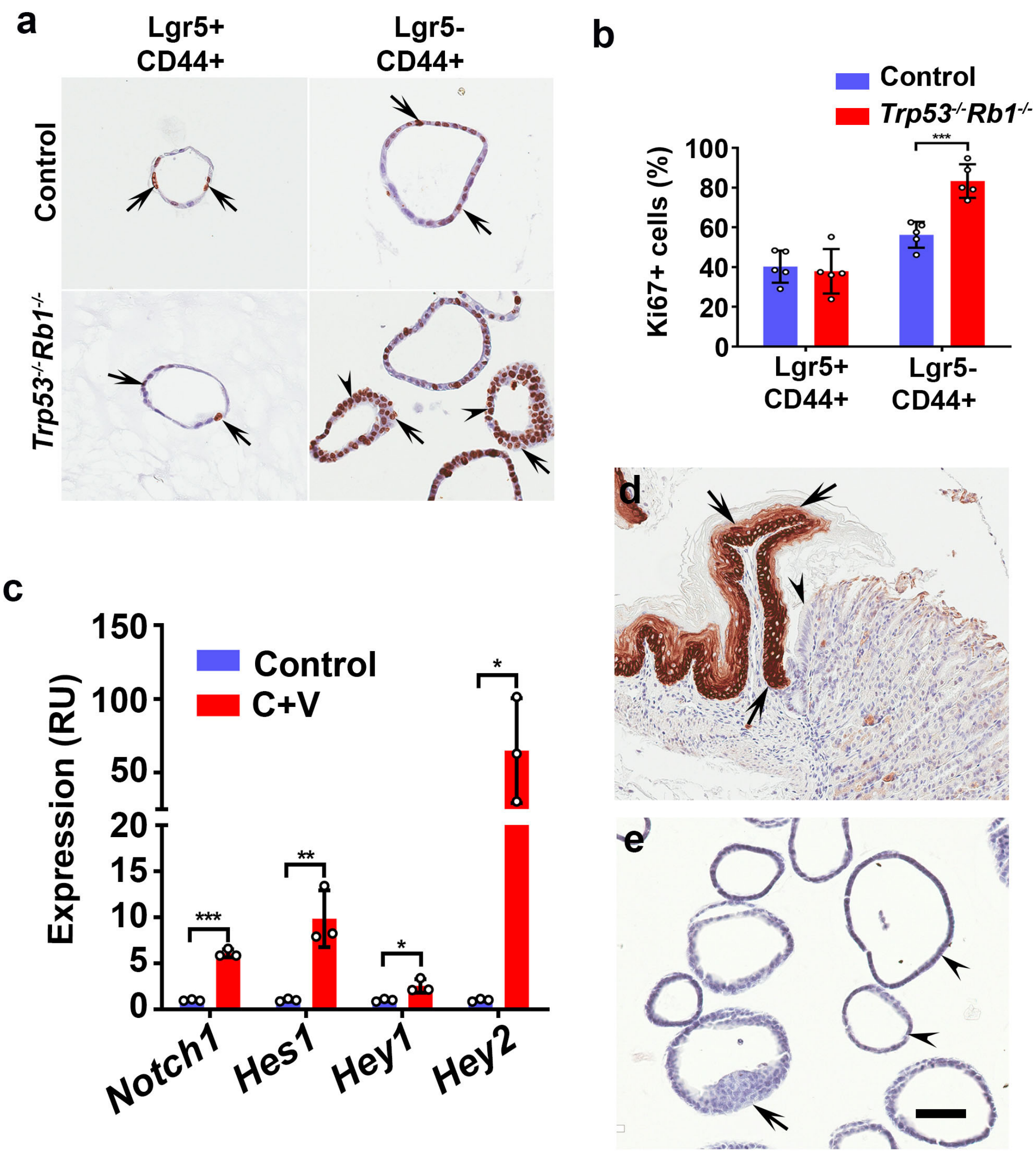

Supplementary Figure 7. Detection of Ki67, Notch signaling, and Keratin 5 (KRT5) in mouse gastric organoids and tissue. a and **b**, Representative image (**a**) and quantification (**b**) of Ki67-expressing cells (arrows in **a**) in Lgr5+CD44+ and Lgr5-Cd44+ organoids before and after CRISPR-*Trp53/Rb1* treatment. (n=5). **c,** The effect of CHIR99021 and VPA on expression of Notch signaling-related genes in SCJ organoids. (n=3). **d,** KRT5 is detected in the squamous epithelium (arrows) but not in the glandular epithelium (arrowhead). **e,** KRT5 is not detected in dysplastic (arrow), or morphologically normal (arrowheads) organoids. Counterstaining with hematoxylin (**a***,* **d,** and **e**). Scale bar in **e** represents 60 µm (**a**), 100 µm (**d**), and 50 µm (**e**). *P<0.05, **P<0.01, ***P<0.001, two-tailed unpaired t-test. All error bars denote s.d. Source data are provided as a Source Data file.

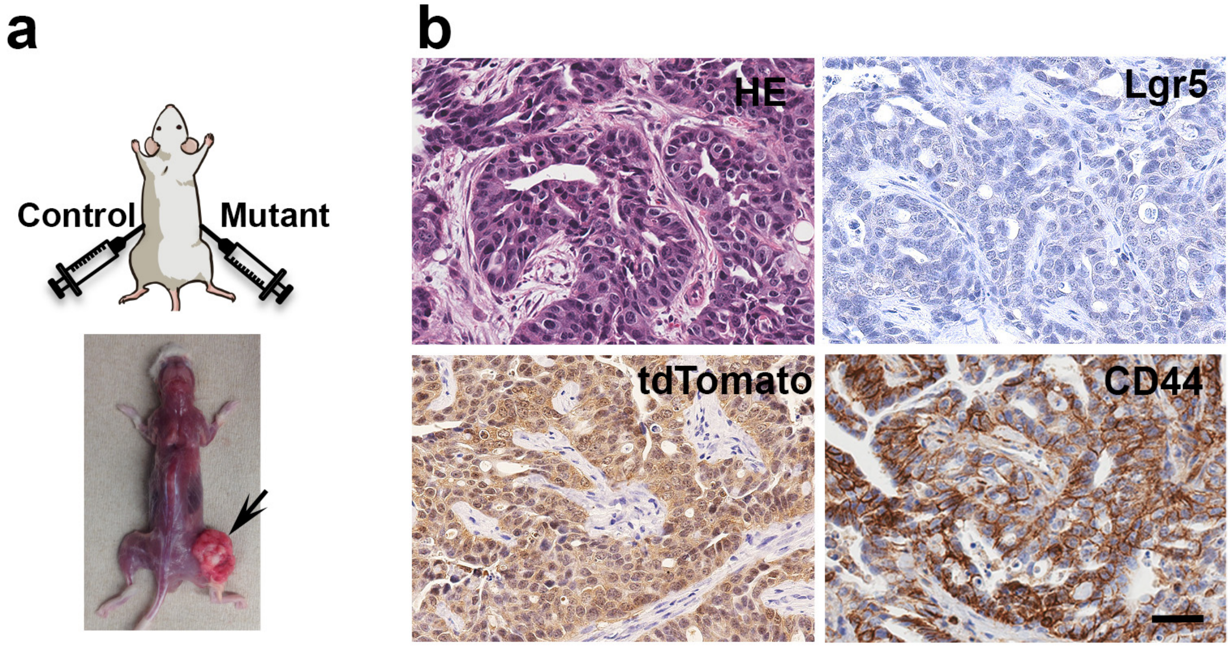

Supplementary Figure 8. Characterization of neoplasms formed from gastric SCJ organoids deficient for *Trp53* and *Rb1*. a and **b**, Gross (**a**) and histological (**b**) images of neoplasm (arrow in **a**) formed from *Lgr5*$^{eGFP\text{-}Ires\text{-}CreERT2}$*Trp53*$^{loxP/loxP}$*Rb1*$^{loxP/loxP}$Ai9 mouse derived and tamoxifen-exposed (mutant) organoids 223 days after s.c. transplantation into NSG mouse. Control, transplanted organoids without tamoxifen induction. Neoplastic cells express tdTomato and CD44 (brown color) but not Lgr5-eGFP (Lgr5). Hematoxylin and eosin (HE, **b**, upper left image). Elite ABC method, counterstaining with hematoxylin (**b**, other images). Scale bar in **b** represents 2 cm (**a**) and 50 µm (**b**).

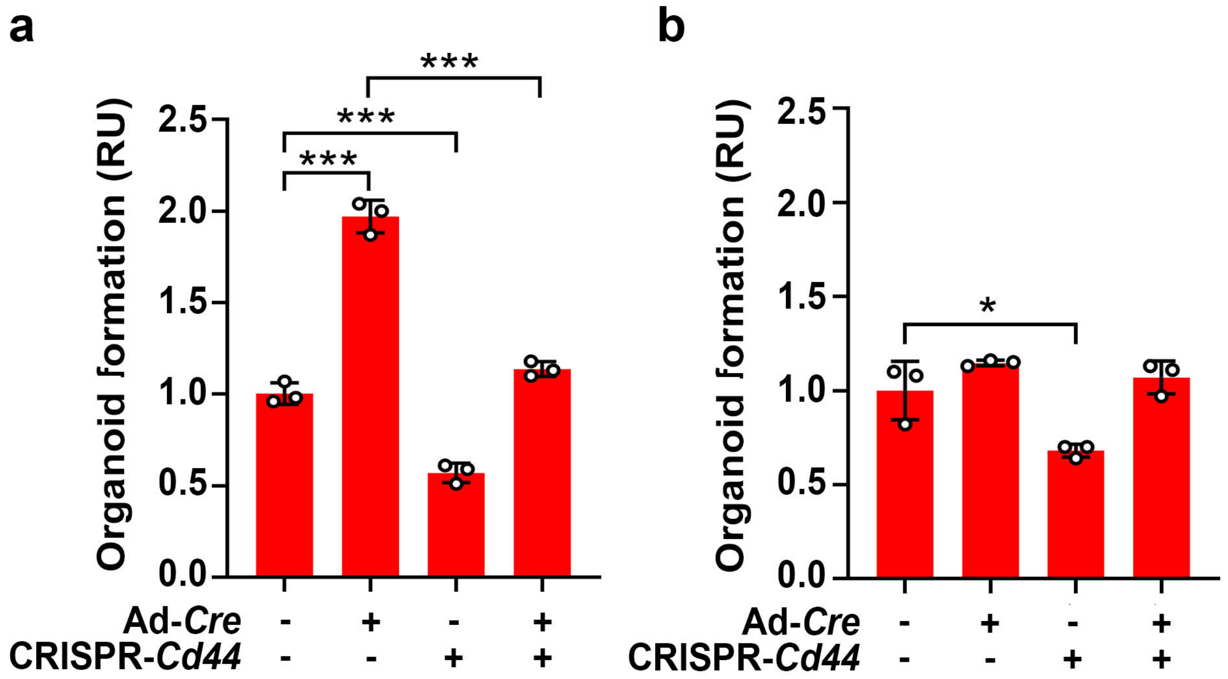

Supplementary Figure 9. Formation of gastric SCJ and antral organoids. a and **b,** The relative number of organoids derived from the gastric SCJ (**a**) and antrum (**b**) of *Lgr5*$^{eGFP\text{-}Ires\text{-}CreERT2T}$*Trp53*$^{loxP/loxP}$*Rb1*$^{loxP/loxP}$Ai9 mice before and after inactivation of *Trp53* and *Rb1* (Ad-*Cre*) and/or CD44 (CRISPR-*Cd44*). The organoid numbers were normalized to control organoids in the same group. n=3. *P<0.05, ***P<0.001, two-tailed unpaired t-tests. All error bars denote s.d. Source data are provided as a Source Data file.

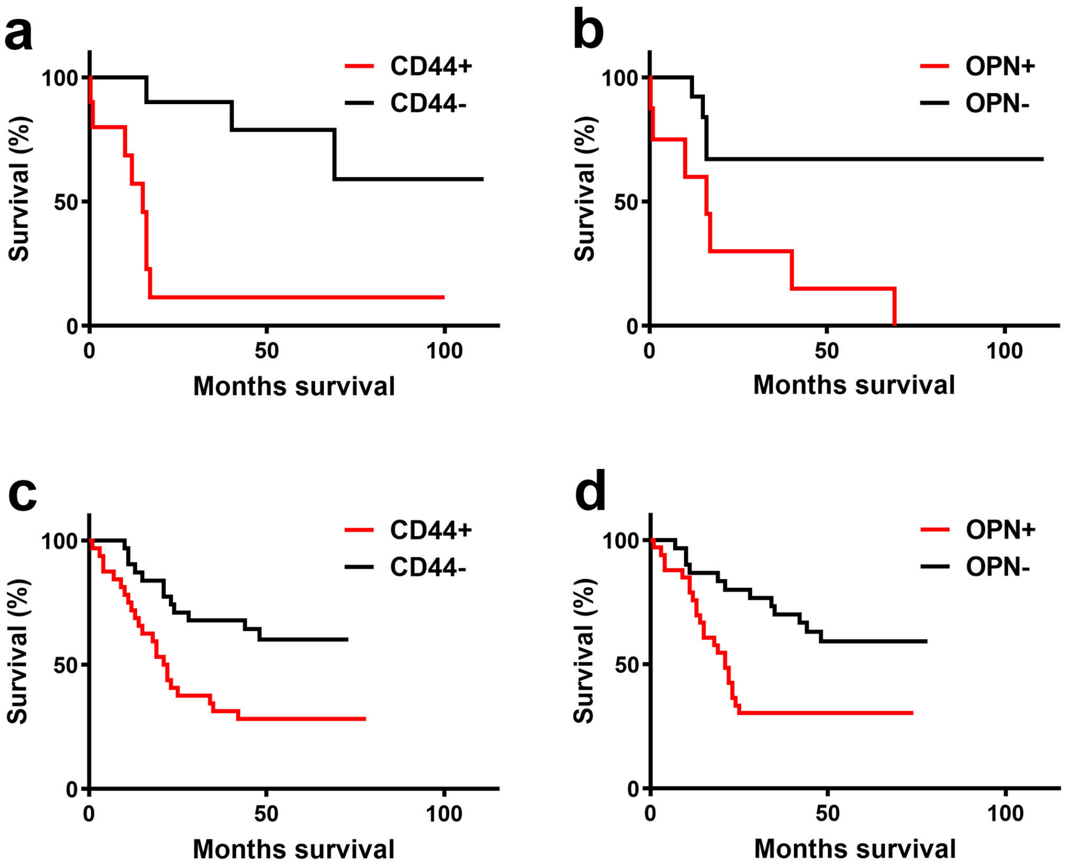

Supplementary Figure 10. Association of CD44 and OPN expression with survival of GEJ cancer patients in two cohorts. a and **b**, Kaplan–Meier survival analysis of GEJ cancer patients (Shihezi University School of Medicine) with CD44 (**a**, CD44+ n=10, CD44- n=11, P=0.0018) and OPN (**b**, OPN+ n=8, OPN- n=13, P=0.0081) expression. **c** and **d,** Kaplan–Meier survival analysis of GEJ cancer patients (US Biomax) with CD44 (**c**, CD44+ n=32, CD44- n=31, P=0.0038) and OPN (**d**, OPN+ n=33, OPN- n=30, P=0.0055) expression. Source data are provided as a Source Data file.

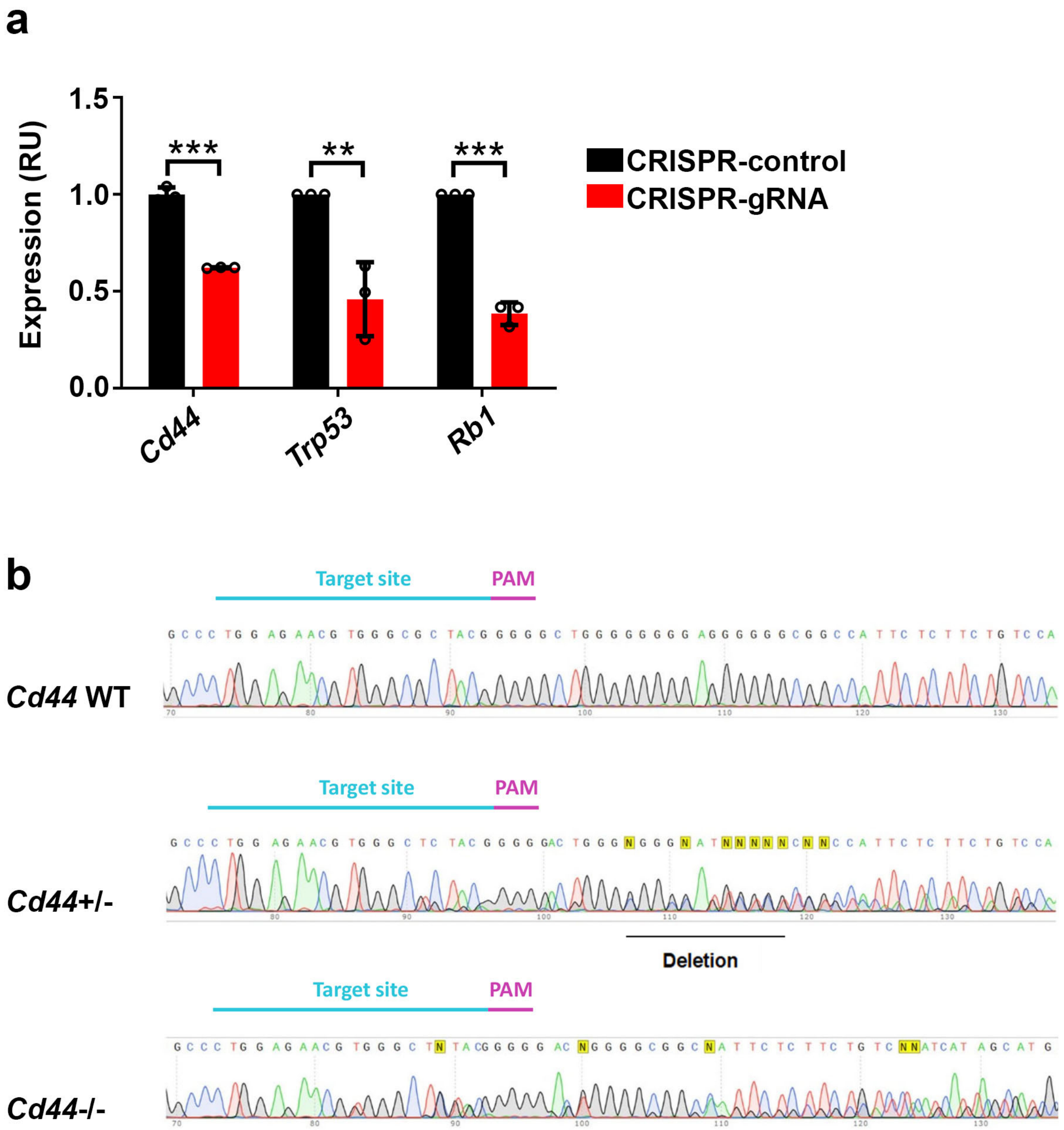

Supplementary Figure 11. Inactivation *Trp53*, and *Rb1*, *Cd44* in gastric organoids using CRISPR/Cas9. a, Relative expression of indicated genes before and after CRISPR-mediated inactivation (n=3 in each group). Normalized to each control group. All error bars denote s.d., **b**, PCR amplification products of the mutated alleles of *Cd44* using primers flanking targeted promoter. Expected indels were confirmed by Sanger sequencing. Out of 38 sequenced samples 10 cells (26%) retained wild-type CD44 alleles, 18 had heterozygous status (47%) and 10 cells (26%) were homozygous for CRSPR mutations. **P<0.01***P<0.001, two-tailed unpaired t-test. All error bars denote s.d. Source data are provided as a Source Data file.

Supplementary Table 1. Clinical characteristics of the patients with gastroesophageal junction cancers (Shihezi University School of Medicine)

Characteristics	No. of cases
**Sex**	
Male	51
Female	7
**Age (mean)***	
< 66	25
< 66	32
**Size***	
< 5 cm	20
< 5 cm	25
**Node metastasis****	
N0	15
N1	40

* Information not available for two cases
**Information not available for 13 cases
***Information not available for two cases

Supplementary Table 2. List of antibodies used for immunostainings

Antigen	Antibody source, catalogue number	Clone	Host	Dilution	Detection system
GFP	NOVUS biological, NB600-303	PC*	Rabbit	1:8000<br>1:1000	Vectastain Elite ABC-HRP Kit<br>Immunofluorescence
CD44	Santa Cruz, sc-18849	IM7	Rat	1:1000<br>1:200	Vectastain Elite ABC-HRP Kit<br>Immunofluorescence
Mucin5AC	Abcam, ab3649	45M1	Mouse	1:500	Mouse on mouse (M.O.M.) detection kit (Vector Laboratories, BMK2202)
H^+K^+-ATPase	MBL international corp., D032-3H	2B6	Mouse	1:500	Mouse on mouse (M.O.M.) detection kit (Vector Laboratories, BMK2202)
Chromogranin A (C-20)	Santa Cruz, sc-1488	PC*	Goat	1:2000	Vectastain Elite ABC-HRP Kit
Pepsinogen C	Abbexa, abx002093	PC*	Rabbit	1:400	Vectastain Elite ABC-HRP Kit
OPN	R&D Systems, AF808	PC*	Goat	1:200<br>1:100	Vectastain Elite ABC-HRP Kit<br>Immunofluorescence
OPN	Sigma, HPA027541	PC*	Rabbit	1:200	Vectastain Elite ABC-HRP Kit
RFP	Rockland Immunochemicals Inc., 600-401-379S	PC*	Rabbit	1:400<br>1:100	Vectastain Elite ABC-HRP Kit<br>Immunofluorescence
BrdU	Abcam, ab2284	PC*	Sheep	1:100	Immunofluorescence
Ki67	Abcam, ab16667	SP6	Rabbit	1:400	Vectastain Elite ABC-HRP Kit
KRT5	Covance, PRB-160P	PC*	Rabbit	1:400	Vectastain Elite ABC-HRP Kit

Anti-rat IgG (Biotinylated)	Vector Laboratories, BA-400	PC*	Rabbit	1:200	Vectastain Elite ABC-HRP Kit
Anti-rabbit IgG (Biotinylated)	Vector Laboratories, BA-1000	PC*	Goat	1:200	Vectastain Elite ABC-HRP Kit
Anti-goat IgG (Biotinylated)	Vector Laboratories, BA-5000	PC*	Rabbit	1:200	Vectastain Elite ABC-HRP Kit
Anti-sheep IgG conjugated with Alexa 594	Invitrogen, A11016	PC*	Donkey	1:200	Immunofluorescence
Anti-rat IgG conjugated with Alexa 488	Invitrogen, A21208	PC*	Donkey	1:200	Immunofluorescence
Anti-rat IgG conjugated with Alexa 594	Invitrogen, A21209	PC*	Donkey	1:200	Immunofluorescence
Anti-goat IgG conjugated with Alexa 488	Invitrogen, A11058	PC*	Donkey	1:200	Immunofluorescence
Anti-rabbit IgG conjugated with Alexa 488	Invitrogen, A21206	PC*	Donkey	1:200	Immunofluorescence
Anti-rabbit IgG conjugated with Alexa 594	Invitrogen, A21207	PC*	Donkey	1:200	Immunofluorescence

PC*: Polyclonal.
